# Prolonged Cell Encapsulation and Gravity‐independent Filamented Light Biofabrication of Muscle Constructs

**DOI:** 10.1002/advs.202512727

**Published:** 2025-09-23

**Authors:** Michael Winkelbauer, Jakub Janiak, Johannes Windisch, Hao Liu, Maria Bulatova, Max Von Witzleben, Hugo Oliveira, Finn Dani, Richard Frank Richter, Nicolas L’Heureux, Ori Bar‐Nur, Michael Gelinsky, Marcy Zenobi‐Wong, Parth Chansoria

**Affiliations:** ^1^ Institute for Biomechanics Department of Health Sciences and Technology ETH Zürich Zürich 8093 Switzerland; ^2^ Center for Translational Bone, Joint and Soft Tissue Research University Hospital and Faculty of Medicine TU Dresden, Fetscherstr. 74 01307 Dresden Germany; ^3^ Univ. Bordeaux, Tissue Bioengineering INSERM U1026 Bordeaux F‐33000 France; ^4^ Laboratory of Regenerative and Muscle Biology Department of Health Sciences and Technology ETH Zurich Schwerzenbach 8603 Switzerland

**Keywords:** aligned tissue, biofabrication, cryopreservation, filamented light (FLight), microgravity

## Abstract

The prospects of fabricating human tissue grafts or models using cell‐laden bioresins in space have garnered significant interest in recent years. While there is tremendous progress in extrusion or light‐based bioprinting in microgravity conditions, printing of aligned tissues (e.g., muscle, tendon, cardiac, etc.) remains a challenge. Furthermore, current photoresin formulations do not allow long‐term cell encapsulation and are difficult to handle in microgravity conditions. In this study, a new gravity‐independent filamented light (G‐FLight) biofabrication system, which can create viable muscle constructs within seconds, is demonstrated. New photoresin formulations based on gelatin methacrylate (GelMA) for encapsulation of primary cells (murine myoblasts) and storage in printing cuvettes for at least a week at 4 °C or ‐80 °C are also demonstrated. The tissues printed in microgravity based on the new formulations exhibit higher cell viability, number of proliferating cells, and higher numbers of myotubes and fusion index compared to control formulations (i.e., GelMA dissolved in phosphate‐buffered saline). Importantly, the microgravity‐printed tissues also featured similar myotube density and fusion index to those printed using the same resins on‐ground. The G‐Flight printing concept, together with the new resins enabling refrigeration or cryopreservation with encapsulated cells, offers a promising solution for biofabrication in space.

## Graphical abstract

A gravity‐independent filamented light (G‐FLight) projection method is presented for rapid biofabrication of anisotropic tissues, demonstrated with muscle constructs as a target application. Specialized photocrosslinkable resins enable refrigeration or cryopreservation with encapsulated cells. Constructs printed in microgravity exhibit myotube maturation equivalent to ground‐based counterparts, underscoring the potential of this platform technology and resin formulations for use in long‐duration space missions.

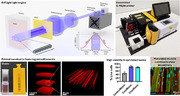

## 1 Introduction

The “space race” during the 1950–70s ushered in a bold and ambitious era of scientific innovation and technological breakthroughs, ultimately pushing the boundaries of what was thought possible in space travel.^[^ ^1^ ^]^ Despite a reduction in crewed space missions thereafter, the spark for interplanetary exploration has persisted amongst the masses, and with the recent development of re‐usable rockets and advanced computing systems, which reduce costs and improve the efficiency of spacecrafts, such an exploration is becoming a tangible possibility.^[^ ^2^, ^3^ ^]^ Sending humans into space, however, would require sustaining lives by understanding the pathophysiological changes to tissues in space (e.g., due the lack of gravity) and developing solutions to repair and regenerate human tissues damaged due to accidents, ageing, and disease.^[^ ^4^, ^5^, ^6^ ^]^ Toward these applications, the field of biofabrication has an important role to play.^[^ ^7^, ^8^ ^]^

There are two strategies for biofabrication in space: 1) The tissues could be biofabricated directly under microgravity conditions, or 2) Tissues could be biofabricated on‐ground and then matured in microgravity.^[^ ^7^, ^8^ ^]^ While fabricating tissues on‐ground and then sending them to space is a logistically and technologically simpler approach, performing biofabrication in microgravity offers the opportunity for printing customized, patient‐specific tissue structures with bespoke spatial distribution of cells and biomaterials catering to the complexity of the target tissues, which is highly relevant for future space exploration.^[^ ^7^ ^]^ The field of biofabrication in microgravity has been largely dominated by studies utilizing extrusion printing, with tissues such as meniscus, bone, and skin successfully fabricated in space using desktop‐based or handheld bioprinting platforms.^[^ ^9^, ^10^, ^11^, ^12^ ^]^ The free‐form material deposition in the absence of gravity allows creation of long filaments and obviates the necessity of support structures, which enables recapitulation of complex 3D tissue structures at a physiological scale.^[^ ^8^, ^13^ ^]^

Notably, biofabrication of anisotropic tissues (such as muscle, nerve, cardiac, etc.) remains a challenge, as current scaffolds created in microgravity lack robust microarchitectural cues for cell orientation and matrix deposition, limiting functional maturation of the tissues. Accordingly, explorations with anisotropic tissues, such as muscle, have been limited to prefabricated tissues contained within perfusion chambers, which are then cultured in microgravity environments.^[^ ^14^, ^15^, ^16^ ^]^ These platforms have offered new insights into disease mechanisms such as sarcopenia in space,^[^ ^14^, ^15^ ^]^ further highlighting the importance of deploying a robust biofabrication approach, which could be used for on‐demand printing of anisotropic tissues directly in space to support long‐term missions.

**Figure 1:**
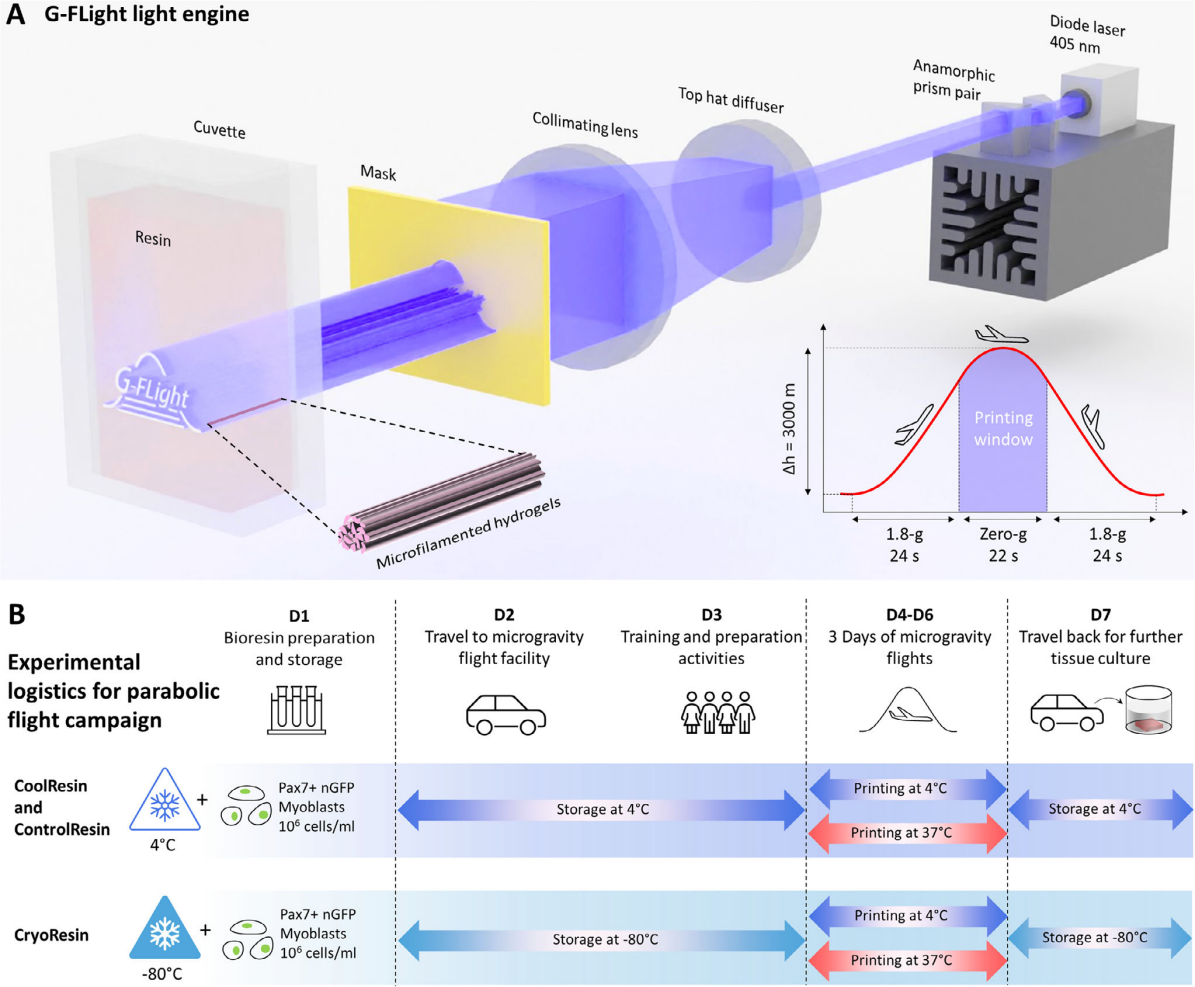
Optical components of G‐FLight printer and resin formulations. A) Illustration of the components of the light engine in the G‐FLight printer and the laser light path (also see Video S1, Supporting Information). Here, the 405 nm laser beam was shaped into an elliptical format using the anamorphic prism pair and later converted into a square profile with a top‐hat diffuser. A plano‐convex lens then collimated the light, which was directed through custom photomasks onto a cuvette filled with the photoresin. This arrangement of components allowed for a robust and minimal form‐factor while allowing rapid FLight printing. B) Logistics of the experiments, which guided the resin storage and printing activities. Of note, this timeline does not include the tissue culture period for 17 days, which accompanied the parabolic flight campaign activities.

We have previously demonstrated FLight biofabrication as a rapid, biocompatible, and scalable approach towards building mm‐scale aligned tissue constructs (on‐ground) mimicking the microarchitecture and matrix components of native muscle,^[^ ^17^ ^]^ nerve,^[^ ^18^ ^]^ and articular cartilage.^[^ ^19^ ^]^ FLight printing relies on the use of light sources, such as a laser, which feature intensity speckles (i.e., the cross‐section of the light beam features regions of high and low intensity).^[^ ^20^ ^]^ Consequently, the photoresin preferentially crosslinks in the regions featuring higher intensity within the speckles, which further leads to optical self‐focusing of the light into the uncrosslinked resin, thereby creating aligned micro‐filamented constructs.^[^ ^17^, ^20^ ^]^ In this work, with skeletal muscle tissues as a target application, we demonstrate a gravity‐independent filamented light (G‐Flight) system for the rapid fabrication (within seconds) of anisotropic tissues under microgravity conditions. The G‐FLight printer builds on our desktop FLight printing approach by deploying a speckled laser light source to create microfilamented constructs. Additionally, the printer incorporates a new optical arrangement enabling a compact and robust device tailored for use in aircraft environments and capable of meeting the vibrational and safety requirements (**Figure** **1**A). As a necessary precursor to future space applications, we have validated the G‐FLight printer within parabolic flights, which recreate several cycles of microgravity lasting 20–22 s, which represents the time window for printing the constructs.

In addition to the considerations for the biofabrication system, the storage and handling of cell‐laden bioresins also needed to be considered. Current bioresin formulations require specialized training for handling in microgravity.^[^ ^21^ ^]^ Here, we posited that the resin handling procedure can be simplified,^[^ ^22^ ^]^ if one could store the cell‐laden resins in refrigeration (4 °C) or cryopreservation (−80 °C) conditions within sealed cuvettes (to ensure sterility), and, at the day of printing, simply thaw the vial (e.g., for the cryopreserved formulations) and print the tissues inside the cuvettes. Accordingly, in this work, we developed new photoresin formulations – CoolResin and CryoResin – which allowed storage with encapsulated cells at 4 or −80 °C, respectively. These formulations were based on gelatin methacrylate (GelMA), as it is one of the most widely used biomaterials in light‐based tissue engineering applications.^[^ ^23^, ^24^, ^25^, ^26^ ^]^ The development of the new formulations was necessary to account for the experimental logistics (illustrated in Figure 1B), which involved several key steps: **1)** Cell encapsulation within the photoresins (i.e., to create the bioresins) followed by loading within the cuvettes (activities performed in the cell culture facility in Zurich, Switzerland), **2)** Establishing the appropriate storage conditions for the cuvettes (storage inside a portable refrigerator (4 °C) or dry ice (−80 °C)), **3)** Transportation to the parabolic flight facility in Bordeaux, France, followed by several days of training and preparation, and then parabolic flight days, during which the printing was carried out, **4)** Storage of the resins back at 4 or −80 °C after each day of printing, **5)** Driving back to the cell culture facility (Zurich, Switzerland), **6**. Retrieving the tissue constructs and culturing them to assess viability, cell differentiation, and maturation characteristics.

As a control formulation (termed as ControlResin henceforth), we used GelMA dissolved in phosphate buffered saline (PBS), to be able to compare the cytoprotective effects and further the tissue maturation for the CoolResin and CryoResin formulations. Furthermore, the GelMA formulations exhibit a thermo‐reversible gelled state at 4 °C and a liquid state at 37 °C, and the properties (elastic modulus, pore size, and porosity, etc.) of the photocrosslinked hydrogels can vary depending on the temperature of the resin before crosslinking.^[^ ^27^ ^]^ Accordingly, we investigated the impact of these two printing temperatures (also indicated in Figure 1B),^[^ ^23^, ^27^ ^]^ to evaluate the resulting differences in constructs post‐fabrication and maturation. We also printed constructs on‐ground in tandem to the microgravity flights and compared them to the microgravity‐printed constructs after maturation. Importantly, we demonstrate that the properties of the tissues fabricated in microgravity are comparable to those fabricated on‐ground, enabling predictability in the scaffold properties (i.e., a gravity‐independent biofabrication system). Finally, we have discussed how our G‐Flight system and optimized resin formulations can be deployed for use in future long‐term space applications for complex anisotropic tissue biofabrication.

## 2 Results

### 2.1 Assembly of the G‐FLight System for Biofabrication Under Microgravity

The optomechanical assembly of the G‐FLight system and additional auxiliary components were designed considering several engineering and logistical constraints. These included: **1)** Size constraint on the available rack space (30(l)×45(w)×30(h) cm) available in the aircraft performing parabolic flight maneuvers (see image of the rack in Figure S1, Supporting Information), 2) The need for an onboard resin refrigeration unit (for storage at 4 °C during the parabolic flights) and heating unit, and **3)** The need to withstand vibrations during transportation and parabolic flight maneuvers. To address these constraints, we excluded sensitive optical components such as a digital micromirror device (DMD) and complex beam‐shaping optics (e.g., telescopic lenses), which we had used in our prior work,^[^ ^17^, ^28^ ^]^ and instead developed a compact and robust light engine featuring 3D printed physical masks for light shaping (illustration in Figure 1A; animation of the light‐path in Video S1, Supporting Information). This system incorporated a 405 nm laser beam, which was initially shaped from an elliptical profile (1 × 5 mm) into a rectangular profile (3 × 5 mm) using an anamorphic prism pair, and later into a square profile (10 × 10 mm) with a 20° divergence angle via a top‐hat diffusor. A plano‐convex lens was then positioned thereafter to collimate the light along a 30 × 30 mm profile (i.e., the maximum permissible size of the image printable using the G‐FLight system). This beam was passed through 3D printed photomasks to shape the light before projection onto a cuvette containing the photoresins (Figure 1A).

**Figure 2:**
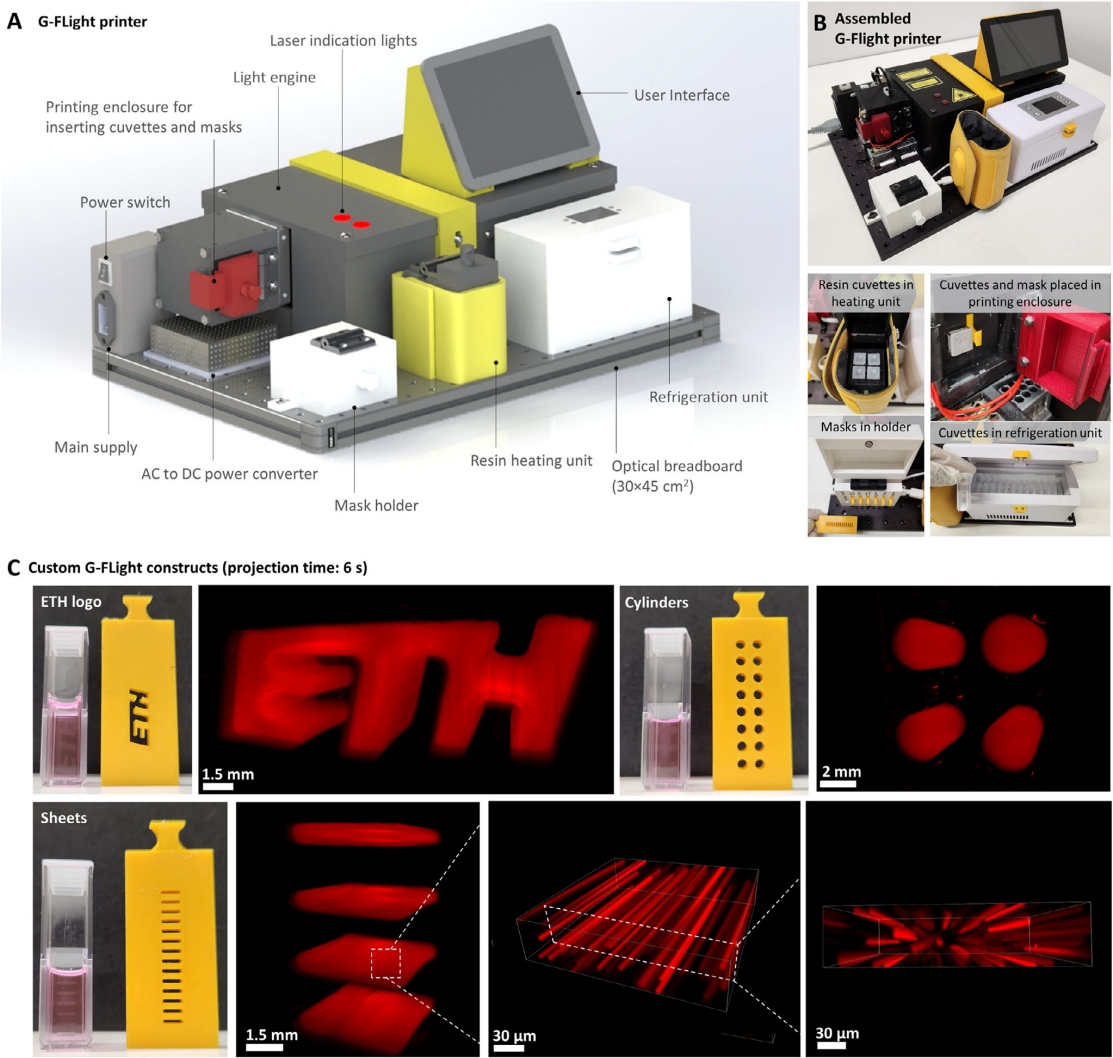
G‐FLight printer and example prints of microfilamented constructs. A) Components of G‐FLight printer contained within a volume of 30(l)×45(w)×30(h) cm. B) The assembled G‐FLight printer and its key components used during the microgravity printing phase. C) Example prints of the ETH logo, cylindrical, and sheet‐like constructs using rhodamine‐labeled resins (ControlResin formulations containing GelMA at 5% w/v within PBS were used for these experiments; see Experimental Section for details). The inset images demonstrate the microfilaments within the G‐FLight printed constructs. The images of the printed constructs were obtained through light sheet microscopy, and images of the microfilaments within the constructs were obtained through confocal microscopy.

An illustration of the assembly of the G‐FLight printer with all its auxiliary components (including the light engine inside an enclosure) is shown in **Figure** **2**A, while the actual assembled printer and the key components are shown in Figure 2B. All the components were mounted on a 30 × 45 cm optical breadboard. The printer housed a refrigeration unit, which stored the bioresins (i.e., cells encapsulated within the photoresins) at 4 °C throughout the duration of the parabolic flight, and a heating unit for heating the resins to 37 °C before printing. This setup allowed recapitulation of the printing temperatures which have been typically used for gelatin‐based resins, where GelMA is in a thermo‐reversibly gelled state at 4 °C, or in a liquefied state at 37 °C.^[^ ^27^, ^28^ ^]^ In the G‐FLight printer, the print parameters (exposure duration and intensity) could be set using the graphical user interface (see Video S2, Supporting Information). Before printing, appropriate masks could be retrieved from the mask holder, and the photoresin‐laden cuvettes retrieved from the heating or refrigeration unit and placed inside the printing enclosure. After printing, the uncrosslinked resin could be washed away (on‐ground) and the printed constructs retrieved from the cuvettes at the cell culture facility (further details in the next section).

Representative prints using photoresins composed of 5% w/v GelMA dissolved in PBS are shown in Figure 2C. Based on the masks used, the light could be selectively shaped to control the cross‐section of the constructs (examples of different masks and the corresponding prints are shown in Figure 2C). Upon reaching its gelation threshold, the illuminated photoresin locally crosslinked into hydrogel constructs featuring micro‐filaments – a characteristic of FLight‐printing – prevalent throughout the length of the constructs.

### 2.2 Development and Characterization of Photoresins for the Encapsulation and Storage of Primary Myoblasts Under Refrigeration and Cryogenic Conditions

**Figure 3:**
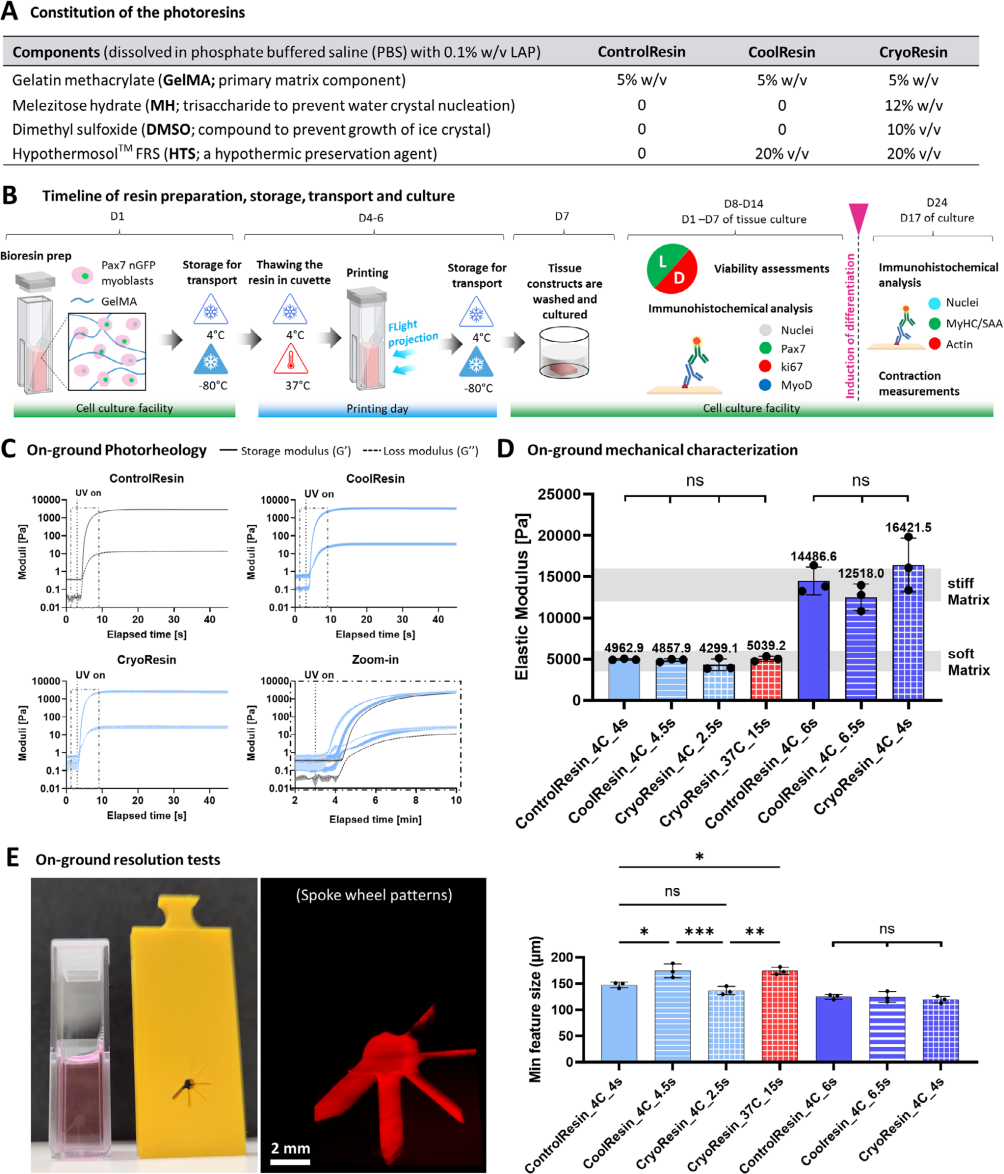
Material constitutions, experimental workflow, and resin printing assessment. A) The components (dissolved in PBS) of the different resin used in the G‐Flight printer. B) General timeline (spanning a total of 24 days) of resin storage, transport, printing, and culture. C) Photorheology of the resin formulations, which demonstrated a rapid photocrosslinking under near UV (405 nm) light exposure. The zoom‐in image shows the collective response of the resin formulations in the dotted region of the individual photorheology curves. D) Elastic modulus (under compression) of the printed constructs, where the exposure duration was changed to increase the crosslinking density in the matrix formulations (the mean values for each group are listed above the individual bars). Through optimization of the light dose during polymerization, we selected specific formulations that were either soft (4–6 kPa) or stiff (12–16 kPa) for engineering the muscle constructs. E) Example prints using the resolution mask with the spoke wheel pattern and the analysis of the minimum feature size obtained using the different resin formulations. The labels 4C or 37C denote the printing temperatures 4 and 37 °C, respectively, while the timing (e.g., 4s, 6s, etc.) denotes the duration of FLight printing within the constructs, respectively. Data represented as mean ± SD (*n* = 3), statistical significance was determined by one‐way ANOVA and is denoted as follows: *refers to *p* < 0.05, ^**^refers to *p* < 0.01, ^***^refers to *p* < 0.001, ns refers to non‐significant difference.

Considering the experimental logistics (previously illustrated in Figure 1B), we developed the CoolResin and CryoResin formulations for storage with encapsulated cells at 4 or −80 °C, respectively, and compared them to ControlResin formulations. The specific compositions of the resins are detailed in **Figure** **3**A. All formulations contained 5% w/v GelMA in PBS with lithium phenyl‐2,4,6‐trimethylbenzoylphosphinate (LAP) as the photoinitiator at 0.1% w/v. Of note, this LAP concentration was optimized from our pilot experiments, where it enabled rapid printing (at each temperature) within the 22 s permissible printing time, while also exhibiting minimal photoabsorption, thereby resulting in uniform stiffness throughout the 4 mm‐long constructs. For CoolResin, we supplemented the PBS solution with Hypothermosol FRS (termed as HTS henceforth), which is a commercially available hypothermic preservation solution.^[^ ^29^, ^30^ ^]^ For CryoResin, the PBS solution was further supplemented (in addition to HTS) with the trisaccharide melezitose hydrate and dimethyl sulfoxide (DMSO), both of which have been used previously in cryo(bio)printing applications to reduce ice crystal formation in GelMA resins during freezing and to improve cell survival.^[^ ^31^, ^32^ ^]^ Melezitose hydrate, analogous to other trisaccharides such as trehalose,^[^ ^33^ ^]^ has been reported to exert cryoprotective effects by replacing water through hydrogen bonding with proteins and membranes, thereby preserving their native conformations.^[^ ^34^, ^35^ ^]^ Melezitose also limits molecular mobility and suppresses lipid phase transitions, thereby stabilizing cellular structures during freezing stress.^[^ ^34^, ^35^ ^]^ In parallel, DMSO acts as a permeating cryoprotectant: at concentrations > 1m it penetrates cell membranes, inhibits freeze‐induced lipid phase transitions, and fusion. Thereby, it stabilizes bilayer organization during cooling and thawing, preserving membrane integrity and enhancing post‐thaw cell survival.^[^ ^36^ ^]^ For the cells, we used satellite cell‐derived Pax7‐nGFP primary myoblasts,^[^ ^33^ ^]^ which express a GFP fluorescent reporter under a Pax7 promoter that is specific for muscle stem cells.^[^ ^37^, ^38^ ^]^ These myoblast cells are known to activate upon injury in vivo, proliferate and differentiate, contributing to regeneration by forming new myotubes or replenishing the stem cell pool.^[^ ^39^, ^40^ ^]^ These cells were especially suited to assessing the effects of the strenuous storage and transport conditions posed by the parabolic flight campaign. They have also been previously demonstrated to form biomimetic myotubes in vitro (upon induction of differentiation through serum withdrawal), exhibiting spontaneous contractions and sarcomere structures.^[^ ^37^, ^41^ ^]^

Pre‐flight on‐ground characterization of the crosslinking kinetics was performed after the storage of the resins for 1 week to account for the logistics of the parabolic campaign (Figure 3B highlights the timeline specific to the resins). Here, the CoolResin and ControlResin formulations were stored 4 °C by simply transferring the sterile resin‐filled cuvettes (with capped lids) in a refrigerator. For CryoResin formulations, the capped cuvettes with the resins were placed in a controlled‐rate freezing unit (Mr. Frosty) containing isopropyl alcohol (IPA), which was in turn placed within a dry ice container, to ensure cooling at 1 °C min^−1^ until reaching −80 °C. Note that the ControlResin and CoolResin formulations were not evaluated for storage at −80 °C as the constructs demonstrated very poor fidelity and low cell viability (< 10%) after printing (pilot results not shown). The photorheological tests confirmed a rapid polymerization of GelMA for different formulations upon exposure to 405 nm light. To isolate the effects of resin additives from the inherent physical crosslinking behavior of GelMA, these measurements were conducted at 25 °C on liquid state (non‐gelled) formulations. Comparative analysis of the crosslinking onset (Figure 3C) indicated faster crosslinking kinetics in the CryoResin formulations compared to the CoolResin and ControlResin, which could be attributed to inhibition of photoinitiator degradation during cryogenic storage. Despite differences in polymerization onset, the final storage moduli after complete crosslinking were comparable, yielding values in the range 2.7–3.4 kPa.

Subsequently, we assessed the effect of photoexposure duration on hydrogel stiffness. Considering ranges used in muscle tissue engineering,^[^ ^42^ ^]^ we classified crosslinked hydrogels exhibiting an elastic modulus of 3–5 kPa as “soft” gels and 12–16 kPa as “stiff” gels (Figure 3D). Here, for the same light engine producing output at 34 mW cm^−2^, we screened a range of exposure times (see Figure S2, Supporting Information). Notably, the resins being printed at 37 °C took longer to polymerize and to achieve crosslinking ranges similar to those printed at 4 °C, which can be attributable to the changes in molecular structure and reactive group availability in the GelMA formulations under cold or heated conditions.^[^ ^27^ ^]^ Considering the printing window during the microgravity phase would be within 22 s, only the CryoResin formulations at 37 °C (termed as CryoResin_37C henceforth) were suitable as they required a 15 s exposure duration. The resulting gels from CryoResin_37C exhibited stiffness values within the soft stiffness range. However, none of the other formulations (when heated to 37 °C) reached the desired stiffness in either the soft or stiff regimes within the available 22 s printing window. Consequently, these formulations were excluded from subsequent cell‐based experiments. From these tests, we obtained optimal exposure times as 4, 4.5, 2.5, and 15 s for ControlResin_4C, CoolResin_4C, CryoResin_4C, and CryoResin_37C, respectively, to cater to the soft stiffness regime (Figure 3C). For the stiff regime, prolonged exposure times of 6, 6.5, and 4 s were used for ControlResin_4C, CoolResin_4C, and CryoResin_4C, respectively (Figure 3D). The optimized light exposure durations were subsequently applied to evaluate the achievable printing resolution using the G‐FLight system. For this, a spoke‐wheel photomask (Figure 3E) was used, and the minimum achievable feature sizes were determined for the different formulations (Figure S3, Supporting Information). In the soft stiffness regime, differences in the minimum achievable feature sizes were observed across formulations, with average resolutions of 147 ± 5.4, 174.5 ± 13.22, 136.6 ± 7.97, and 174.1 ± 6.86 µm for ControlResin_4C, CoolResin_4C, CryoResin_4C, and CryoResin_37C, respectively (Figure 3E). Notably, the constructs within the higher stiffness regime for each formulation exhibited a smaller minimum attainable feature size than those within the lower stiffness regime, while no significant differences in resolution were found amongst the formulations (minimum feature size ≈120 ± 5 µm). The inability to achieve smaller feature sizes with lower exposure duration is attributable to the spreading of light dose within the resin due to optical blurring and free radical diffusion, which necessitates the use of higher optical doses for smaller features. This is a known constraint with laser‐based printing techniques such as tomographic printing, where the optical doses need to be digitally tuned to account for the different feature sizes in an object.^[^ ^43^ ^]^

Before characterizing the microarchitecture of the resin formulations and the characteristics of cells therein, we conducted pilot tests to screen the formulations for their ability to maintain shape fidelity during long‐term culture. This was essential to be able to later study the muscle maturation characteristics. Accordingly, for these experiments, we encapsulated Pax7‐nGFP primary myoblasts and cultured them for one week within both soft and stiff gels for the different formulations. Here, the gels from the “soft” regime, except for the CryoResin_37C formulation, exhibited substantial change in their overall morphology, including warping and shrinking due to cell‐matrix interactions (Figure S4, Supporting Information). The maintenance of the fidelity for the CryoResin_37C formulation, despite being in the soft regime, could be attributed to the prolonged exposure duration (15 s) for these formulations, which can lead to non‐specific crosslinking due to a combination of optical scattering and free radical diffusion.^[^ ^44^ ^]^ The ramifications of non‐specific crosslinking in the CryoResin_37C formulation will be evident in the subsequent studies. Consequently, further microarchitectural characterization and long‐term tissue maturation were limited to gels crosslinked to the stiff regime and the CryoResin_37C formulation, where no morphological alterations were observed during the pilot cell culture tests (Figure S4, Supporting Information).

### 2.3 On‐Ground Assessment of the Microfilament Characteristics and Cell Survival Within the Selected Resin Formulations

**Figure 4:**
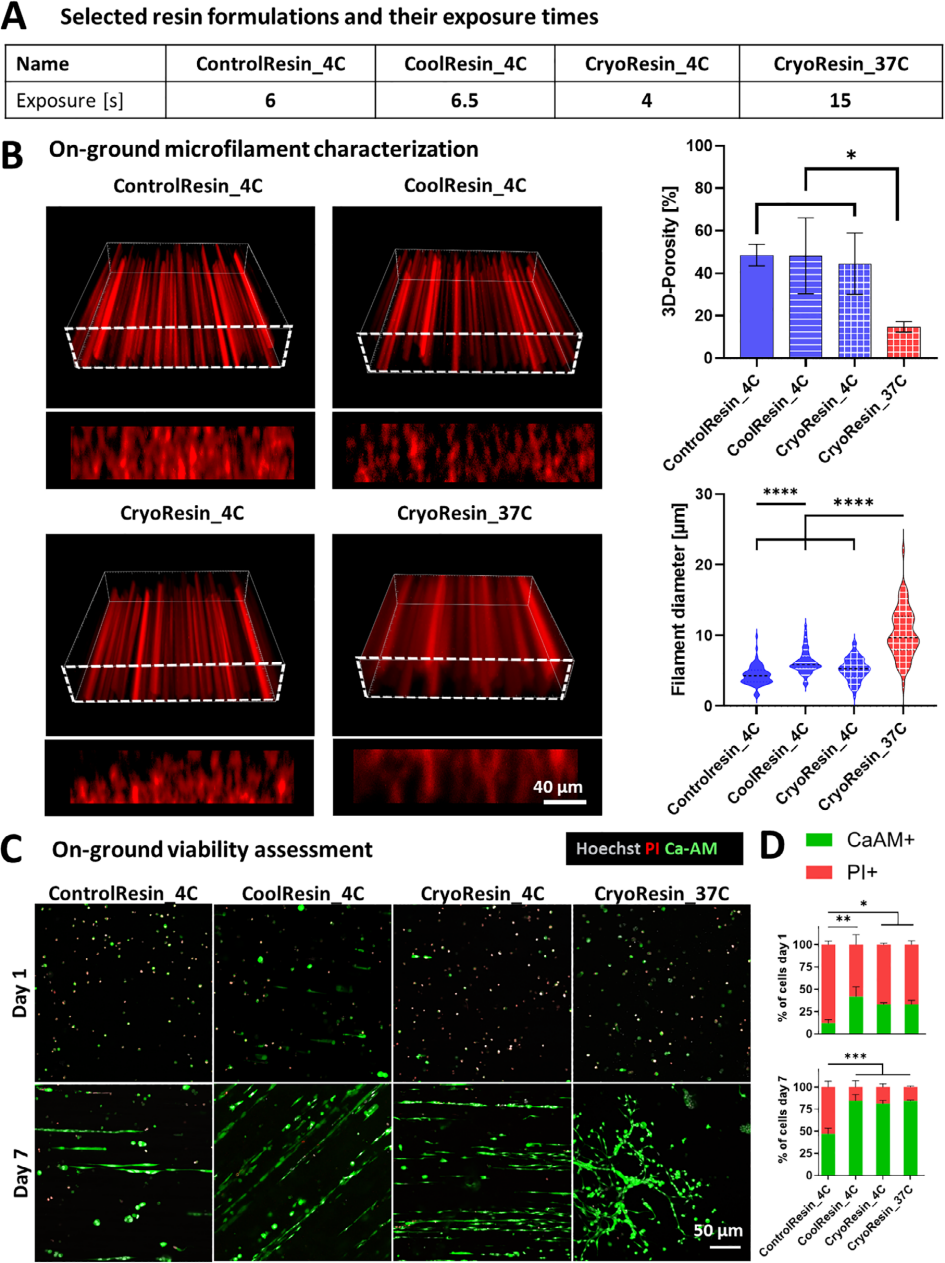
On‐ground pilot assessments of microfilament characteristics and cell survival within selected resin formulations. A) Naming convention (used henceforth) of selected resin formulations and their exposure duration. B) Microfilament distribution within the printed constructs using the selected resin formulations and the analysis of the microfilament size and porosity. C) Microscopic images of the cells stained with propidium iodide (PI) and Calcein AM. D. Analysis of the percentage of cells stained with Calcein AM and PI on days 1 and 7 of culture. Data represented as mean ± SD (*n* = 3), statistical significance was determined by one‐way ANOVA and is denoted as follows: *refers to *p* < 0.05, ^**^refers to *p* < 0.01, ^****^ refers to *p* < 0.0001, ns refers to non‐significant difference.

The G‐FLight system used the FLight concept for the biofabrication of highly aligned microfilamented constructs. Importantly, the microarchitecture of FLight constructs is not only influenced by the fabrication technique (i.e., the light‐engine), but also by the properties of photoresins used (e.g., refractive indices of the resin and concentration, etc.).^[^ ^17^ ^]^ For the selected resin formulations (**Figure** **4**A) obtained after initial tests assessing construct morphology in culture (Figure S4, Supporting Information), we performed confocal imaging to characterize the microfilament distribution within the constructs (Figure 4B). Similar to the previous tests, the samples were stored for 1 week at 4 °C (for CoolResin and ControlResin) or −80 °C after controlled‐rate freezing (for CryoResin).

All resin formulations printed at 4 °C (i.e.,: in their solid state) displayed comparable 3D‐porosity of approximately 50%, with similar distribution of microfilament diameters ranging from 1.5 to 12 µm. Specifically, the porosity (i.e., void space in the 3D constructs) of constructs printed at 4 °C were 48.6 ± 5.1%, 48.2 ± 17.8% and 44.5 ± 14.5% and the microfilament diameters were 4.3 ± 1.4 µm, 6.2 ± 1.7 µm and 5.2 ± 1.6 µm for ControlResin_4C, CoolResin_4C, and CryoResin_4C, respectively (see naming conventions in Figure 4A). The Cryoresin_37C formulation (i.e.,: in its liquid state) exhibited a significantly lower porosity of 14.8 ± 2.5% and a significantly broader distribution of microfilament diameters ranging from 2.9 to 22.1 µm with a diameter of 10.3 ± 3.5 µm. This low porosity and high variability in the microfilament diameters within CryoResin_37C formulation could be attributed to the prolonged exposure duration (15 s) and non‐specific crosslinking as described in the previous section.

After characterizing the microfilaments in the constructs, we encapsulated Pax7‐nGFP primary myoblasts in the selected resin formulations at 10⁶ cells mL^−1^. The bioresins were transferred into cuvettes and subjected to storage conditions specific to each resin: 4 °C for ControlResin_4C and CoolResin_4C, or –80 °C via a controlled‐rate freezer for CryoResin_4C and CryoResin_37C. Considering the actual printing timeline (Figure 1B), after 4 days of storage, the cryopreserved cuvettes were rapidly thawed by immersion in a 37 °C water bath up to the level of the resin, until completely thawed and transferred to 4 °C for 2 h before light exposure and subsequent fabrication of rectangular cell‐laden constructs (4 × 1 mm) using the G‐Flight printer. This step allowed emulating the conditions of the actual microgravity Flight campaign, where the cuvettes needed to be prepared before the flight and then placed in the onboard refrigerator within the G‐Flight printer to be used during the parabolic flight maneuvers. The ControlResin_4C, CoolResin_4C and CryoResin_4C were printed at 4 °C, while the CryoResin_37C were liquefied in the cuvettes using the onboard heating unit at 37 °C shortly before the printing. Following the printing procedure, all cuvettes were transferred to 4 °C for an additional 2 h to emulate aircraft conditions. Finally, to simulate transportation of the cuvettes from the parabolic flight facility (in Bordeaux) to the cell and tissue culture laboratory (in Zurich), the cuvettes containing the bioresins were stored under their respective conditions: CryoResin‐based samples were re‐frozen to −80 °C using controlled‐rate freezing, and ControlResin and CoolResin‐based samples were refrigerated at 4 °C for an additional 3 days. Constructs were then rapidly thawed (in a 37 °C water bath), washed extensively with PBS, and cultured in myoblast expansion medium for 7 days. Cell viability was assessed on days 1, 4, and 7 using CalceinAM (CaAM) and propidium iodide (PI) staining.

The selected images of the constructs from CaAM and PI staining are shown in Figure 4C, and the corresponding data analysis is shown in Figure 4D.^[^ ^36^ ^]^ On Day 1, the highest proportions of viable (i.e., CaAM⁺) cells were observed in the tailored resin formulations, including the CoolResin_4C (41.7 ± 11.2 %), CryoResin_4C (33.2% ± 1.8 %), CryoResin_37C (33.3% ± 4.3%), which were significantly higher than the ControlResin_4C formulations (12.1 ± 4.1). The poor viability in the ControlResin_4C can be attributed to the absence of any HTS, which would otherwise act as a hypothermic preservation agent. Interestingly, on day 1, the formulations also contained some double‐positive cells (CaAM⁺/PI⁺), indicating membrane‐compromised cells (Figure 4C), which is consistent with literature.^[^ ^31^ ^]^ This could explain the less than ideal viability (we aimed for >80% viability on Day 1) observed on Day 1 for the CoolResin and CryoResin formulations. Nevertheless, by day 7, the proportion of viable cells had increased significantly, suggesting cellular recovery. Viability was assessed as 46.7% ± 6.6 %, 84.4% ± 7.1%, 81.2% ± 3.7 %, and 84.2% ± 1.2 % for ControlResin_4C, CoolResin_4C, CryoResin_4C, and CryoResin_37C, respectively. The viability for the CoolResin and CryoResin formulations on Day 7 was significantly higher than the ControlResin formulations.^[^ ^45^ ^]^

### 2.4 Microgravity Printing and Assessment of Myoblasts Differentiation Within the Tissue Constructs

Our on‐ground analysis of the microarchitecture and cell survival allowed us to confirm the suitability of the resin formulations, in use with the G‐FLight system, for subsequent printing in microgravity. For these experiments, the cells were encapsulated in the resins and loaded into cuvettes similar to the previous cell study. The cuvettes were then stored under their respective conditions at 4 °C (portable refrigerator) for the ControlResin and CoolResin formulations, or at −80 °C (using the controlled rate freezing procedure as described previously) for the CryoResin formulations.

**Figure 5:**
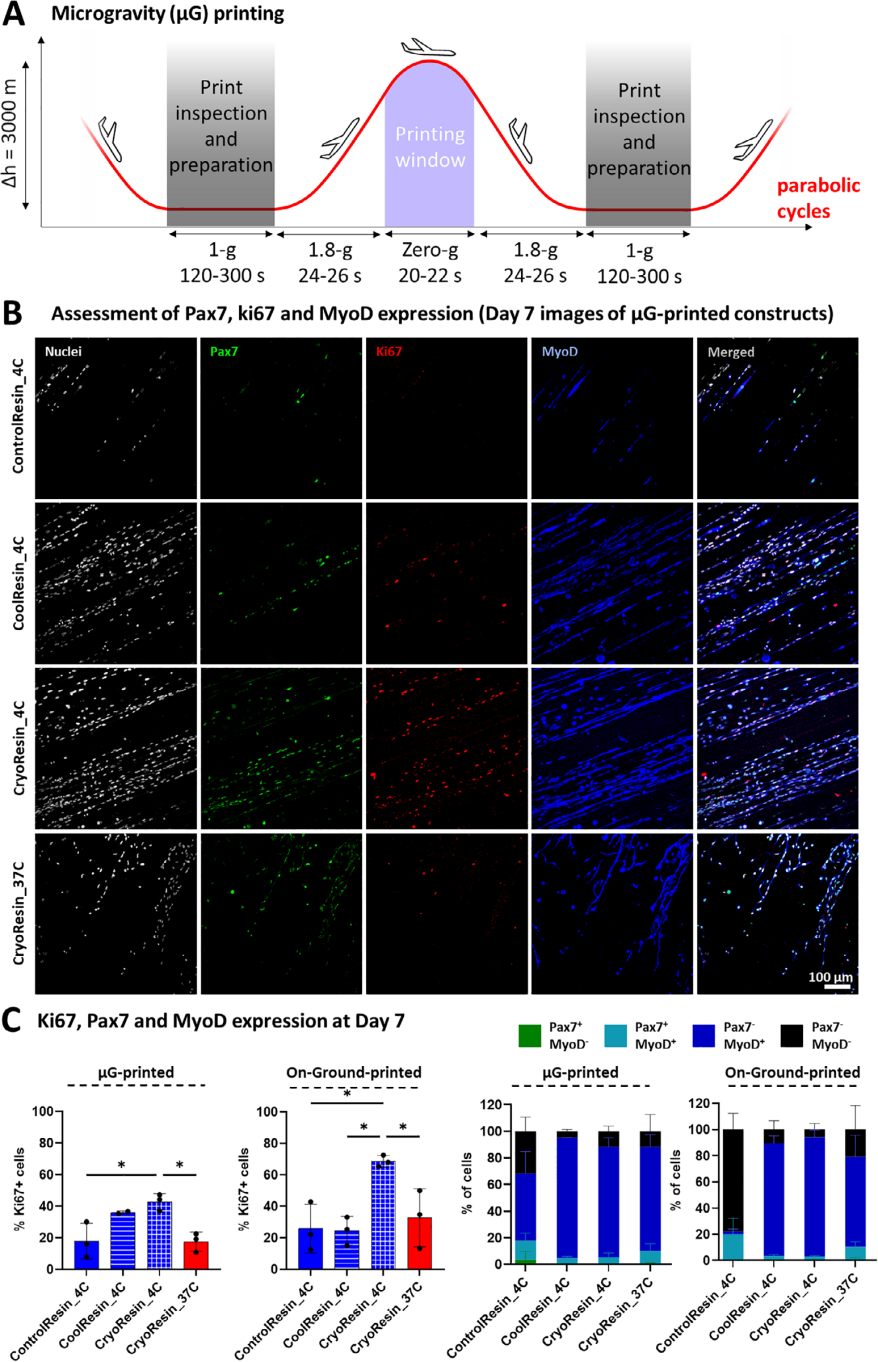
Microgravity printing activities and assessment of growth characteristics within the microgravity‐printed constructs. A) The parabolic cycles (30 in total) consisted of several phases, where the cuvettes were retrieved from the refrigeration or heating unit, inspected, and loaded into the printer during the 1 g (i.e., normal gravity) phase, and the prints were executed during the 0 g (i.e., microgravity) phase. B) Microscopic images of the different resin formulations printed in microgravity (i.e., µG‐printed constructs) demonstrating Pax7^+^ (green), Ki67^+^ (red) and MyoD^+^ (blue) cells (nuclei are grey) at day 7. C) Analysis of the % of Ki67^+^ cells and the myoblast differentiation state through the expression of Pax7^+^ and MyoD^+^ cells within the µG‐printed or On‐Ground‐printed constructs after 7 days of culture. Data is represented as mean ± SD (*n* = 3); statistical significance between groups was determined using one‐way ANOVA, where *refers to *p* < 0.05.

On each flight day, the cuvettes were retrieved and either directly transferred to the onboard refrigeration unit in the G‐FLight printer (for ControlResin and CoolResin), or first rapidly thawed in a water bath at 37 °C (for CryoResin) before transferring to the onboard refrigerator. Before parabolic maneuvers, the photomask (rectangular slits: 4 × 1 mm (w × h)) and the corresponding cuvette were installed in the printer. Each parabolic cycle (illustrated in **Figure** **5**A) was initiated by a steep ascent, during which the aircraft experienced ≈1.8 g for ≈24 s. Shortly before reaching the apex of the parabola, the crew signaled the onset of the microgravity phase (≈0 g, ≈22 s), during which the printing was performed while the aircraft was in freefall. After this zero‐g phase, the aircraft entered a steep descent phase (≈1.8 g for ≈24 s), completing one full parabolic flight maneuver. Between maneuvers, the 120–300 s interval in steady flight (1 g phase) allowed for inspection of printed samples and preparation of the next print (i.e., loading a new cuvette). A recording of preparation, printing, and inspection during the different phases of a parabolic flight maneuver is shown in Video S3 (Supporting Information).

Upon return to ground, the cuvettes were transferred back to their respective storage conditions (i.e., fridge or controlled‐rate‐freezer) until they were transported back to the cell culture laboratory (in Zurich) at the end of the parabolic flight campaign. In the cell culture facility, the cuvettes with the printed constructs were thawed and washed thoroughly with PBS, followed by incubation in myoblast expansion medium. All samples were maintained in expansion medium for 7 days, after which the culture was switched to differentiation medium for 10 days to induce myoblast differentiation into myotubes. At the end of the expansion and differentiation periods, the constructs were fixed and processed using immunofluorescent staining.

At the end of the expansion period (i.e., day 7), we analyzed cells for the expression of Ki67 (i.e., proliferating cells), Pax7‐nGFP (i.e., satellite cells with myogenic progenitor and self‐renewal properties), and MyoD (i.e., cells differentiating towards myogenesis) (Figure 5B). A uniaxial cell alignment along the microfilament direction was present in all constructs printed at 4 °C in microgravity (µg). In contrast, the alignment was disrupted in the CryoResin_37C formulation, which could be attributed to non‐specific crosslinking within these formulations, reducing porosity and limiting cell guidance cues from the microfilaments (further quantification of the alignment is presented in the next section). Quantification of proliferating cells (Ki67⁺) within the µG‐printed constructs revealed that the CoolResin_4C and CryoResin_4C supported the highest levels of proliferation, with 36.1% ± 1% and 42.8% ± 5.2% Ki67⁺ cells, respectively (Figure 5C). In contrast, ControlResin_4C and CryoResin_37C showed markedly lower proliferation, with only 18% ± 11.3% and 17.6% ± 6% Ki67⁺ cells, respectively. The significantly lower amounts of Ki67⁺ cells in the ControlResin_4C group can be attributed to the absence of components for hypothermic preservation or cryopreservation, which caused reduced cell viability and overall cell count. The corresponding lower amounts of Ki67⁺ cells in the CryoResin_37C can be attributed to the low porosity of the matrix, which can limit cell proliferation and impede alignment. Importantly, we also compared the µG‐printed constructs with “On‐Ground” printed constructs, which were printed in normal gravity using the same cell passage, resin compositions, and light doses as the microgravity prints.^[^ ^17^ ^]^ Within the On‐Ground‐printed constructs (microscopic images of selected constructs are shown in Figure S5, Supporting Information), the CryoResin_4C group demonstrated higher % of Ki67⁺ cells, while other groups were similar (Figure 5C). This trend is similar to that observed in µG‐printed constructs, where the CryoResin_4C condition exhibits the highest proportion of Ki67⁺ cells.

To further characterize the cell state, we assessed MyoD expression and used Pax7‐nGFP fluorescence signal to identify muscle stem/progenitor cells (Pax7^+^/MyoD^−^ and Pax7^+^/MyoD^+^) as well as committed cells (Pax7^−^/MyoD^+^). Although no statistically significant differences in MyoD or Pax7 expression were detected between groups, the observed trends were consistent between the µG‐printed and On‐Ground‐printed constructs (Figure 5C). At day 7, Pax7 expression had decreased, likely due to continued proliferation and increased cell densities before the switching to the differentiation medium. Nevertheless, the increased number of proliferating cells and myogenic commitment of the cells within the CoolResin and CryoResin formulations demonstrated the promising potential of these formulations in creating contractile muscle constructs, as explored in the subsequent studies. The overall similarity in the trends for the Ki67, Pax7, and MyoD expression between the µG‐printed and On‐Ground‐printed constructs demonstrated the gravity‐independent nature of the printing process.

### 2.5 Assessment of Myotube Characteristics in the Microgravity‐Printed Constructs After Maturation

**Figure 6:**
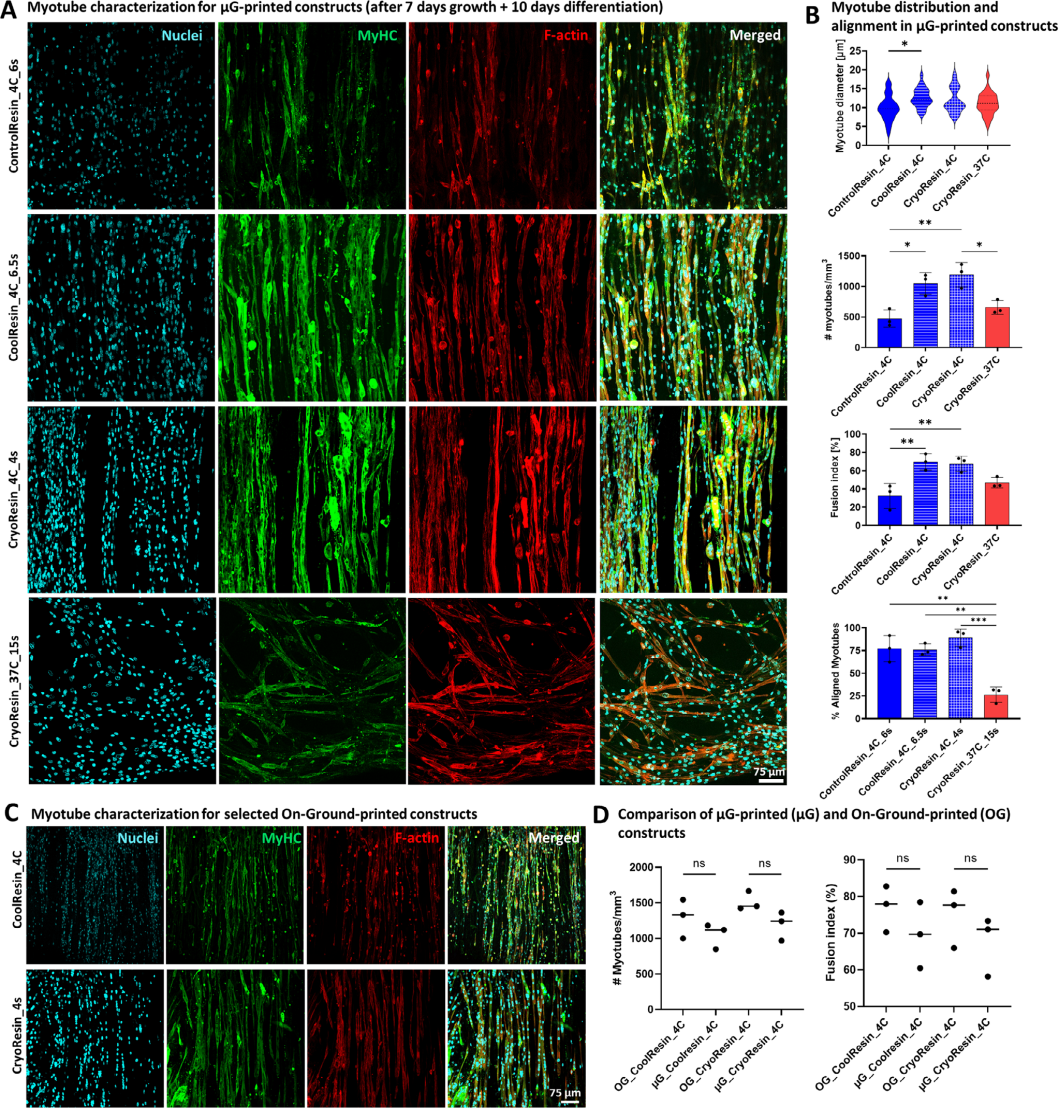
Myotube characteristics in the microgravity (µG)‐printed constructs and comparison with on‐ground constructs. A) All µG‐printed constructs demonstrated myotube formation after 17 days in culture (1 week of growth + 10 days of differentiation). B) The constructs featured an average myotube diameter between 10 and 15 µm, with the Controlresin_4C formulations demonstrating the lowest average diameter amongst the formulations. The number (#) of myotubes mm^−3^ and the fusion index were the highest within the CoolResin_4C and CryoResin_4C formulations) and porosity of the constructs (compared to the Cryoresin_37C formulations). The % aligned myotubes within the constructs was defined as the proportion of myotubes oriented within ±10° of the dominant peak orientation. Notably, the alignment of the myotubes in all formulations except for the Cryoresin_37C formulation was uniaxial, i.e., in the direction of the microfilaments. Data represented as mean ± SD (*n* = 3), statistical significance was determined by one‐way ANOVA and is denoted as follows: ^*^refers to *p* < 0.05, ^**^refers to *p* < 0.01. C) Staining for myosin heavy chain within the constructs printed On‐Ground after maturation (7 days expansion + 10 days differentiation). D) Comparison of µG‐printed (µG) and On‐Ground‐printed (OG) printed constructs using the CoolResin_4C and Cryoresin_4C formulations shows no difference in the myotube density and fusion index within the matured constructs for each formulation.

As mentioned previously, the differentiation of the constructs was initiated on day 7 of tissue culture by switching from expansion medium to differentiation medium. Myogenic maturation was assessed after an additional 10 days in culture via immunofluorescent staining for myosin heavy chain (MyHC) and filamentous actin (F‐actin). Staining confirmed the fusion of myoblasts into multinucleated myotubes across all groups (**Figure** **6**A). The myotube diameter for ControlResin_4C, CoolResin_4C, CryoResin_4C, and CryoResin_37C was 9.9 ± 3.3 µm, 12.6 ± 2.6 µm (significantly higher than that for ControlResin_4C), 11.9 ± 3.3 µm, and 11 ± 2.9 µm, respectively. Myotube density was the highest in CoolResin_4C and CryoResin_4C at 1.15 ± 0.2 × 10^3^ and 1.2 ± 0.2 × 10^3^ myotubes mm^−3^, respectively, while significantly lower densities were observed in ControlResin_4C (0.5 ± 0.1 × 10^3^ myotubes mm^−3^) and CryoResin_37C (0.7 ± 0.1 × 10^3^ myotubes mm^−3^). These differences can be explained by the significantly higher initial cell numbers before differentiation in the former two groups (previously highlighted in Figure 5C). Using the MyHC signal, we further calculated the fusion index (i.e., the number of nuclei within myotubes divided by the total number of nuclei) and found similar trends, with the highest fusion indices in the CoolResin_4C and CryoResin_4C constructs (Figure 6B). We also performed analysis of the alignment of the myotubes within the constructs (Figure S6, Supporting Information). The summarized data for the percentage of aligned myotubes falling within ±10° of the dominant alignment peak is presented in Figure 6B. All groups except the CryoResin_37C formulations demonstrated a uniaxial alignment of >75% of myotubes on average. The disrupted alignment of the myotubes in the CryoResin_37C formulations can be attributed to the low porosity of the matrix (as also previously highlighted in the studies quantifying the Ki67 and MyoD expression within the constructs). Collectively, these results indicate that CoolResin_4C and CryoResin_4C supported the most robust myogenic differentiation, alignment and maturation. Notably, these groups also exhibited myotube characteristics comparable to those printed On‐Ground (Figure 6C,D). These groups were therefore analyzed in the subsequent study for presence of sarcomere structures and their electrical response.

### 2.6 Assessment of Sarcomere Structures and Contractile Response of Selected Microgravity‐Printed Constructs After Maturation

**Figure 7:**
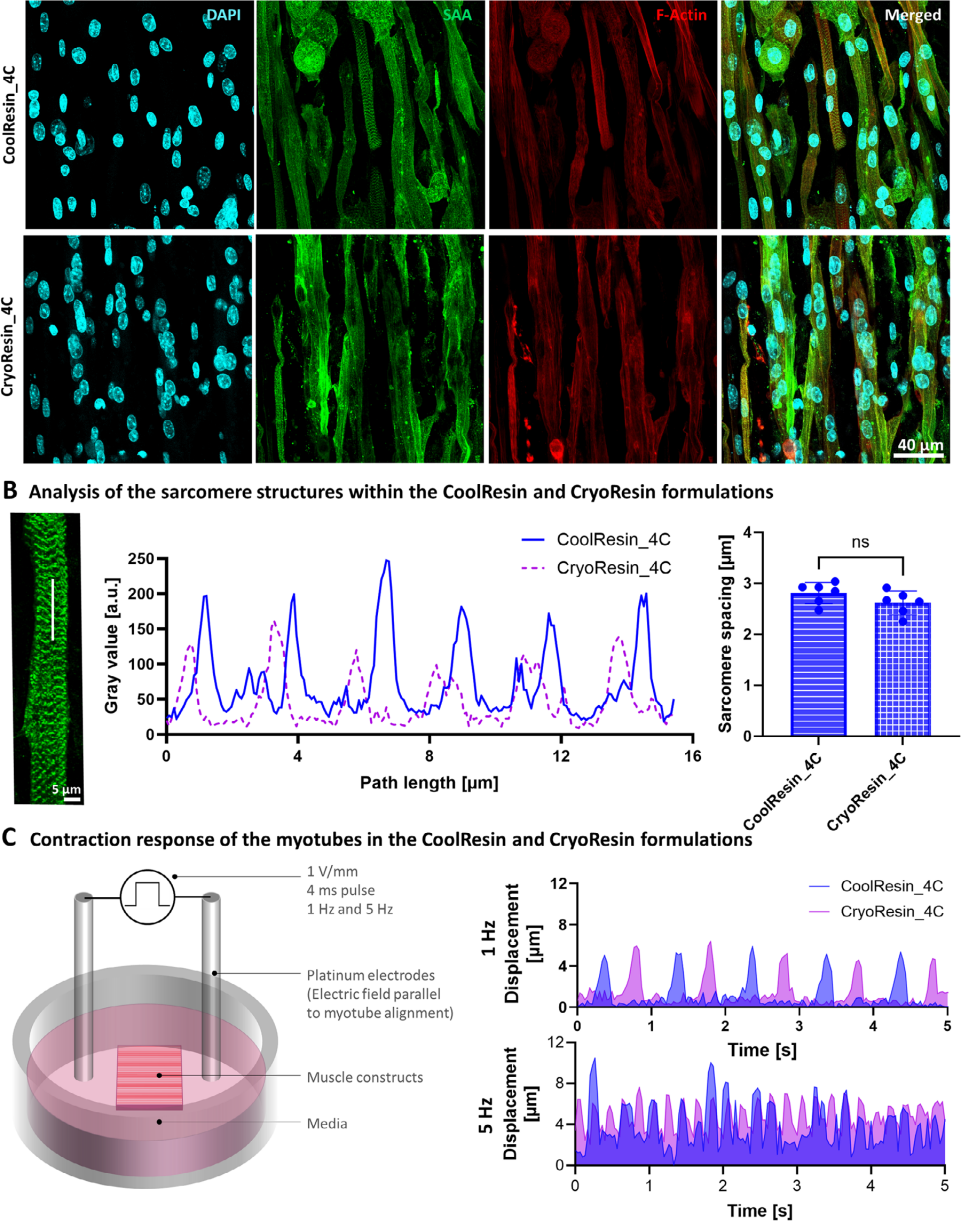
Characterization of the sarcomere structure and contractile response within the microgravity‐printed muscle constructs. A) The constructs made from Coolresin_4C and CryoResin_4C formulations demonstrating sarcomeric alpha actin staining and regularly interspaced sarcomere structures. B) Analysis of the sarcomere structures (gray values are plotted along a selected line (white color) over the sarcomere images) from both formulations, exhibiting a consistent sarcomere spacing of ≈2.8 µm within the constructs. C) The printed constructs demonstrated spontaneous contractility and synchronization to external electrical stimulus (apparatus) providing 4 ms electrical pulses of 1 V mm^−1^ amplitude at 1 and 5 Hz (also see Videos S4 and S5, Supporting Information). Note that the initiation of the excitation was shifted by approximately half the frequency to be able to observe a construct response for each formulation more clearly. Here, the muscle fibers within the constructs made using both CoolResin_4C and Cryoresin_4C formulations demonstrated similar contraction amplitude and synchronization to external stimulus.

Based on the previous findings, we selected the best‐performing conditions (CoolResin_4C and CryoResin_4C) for further analysis of functional muscle maturation within the microgravity (µG)‐printed constructs. Spontaneous myotube contractions were observed as early as day 12 of culture (i.e., day 5 of differentiation) in these two formulations. Constructs were stained for sarcomeric α‐actinin (SAA) to visualize the basic contractile units of skeletal muscle, the sarcomeres (**Figure** **7**A). Morphological analysis revealed well‐aligned, striated myotubes with regular sarcomere spacing of ≈2.7 µm (Figure 7B), which lies within the range typically found in mouse muscle.^[^ ^46^ ^]^

To assess functional contractility, we evaluated the response of selected constructs from the CoolResin_4C and Cryoresin_4C to external electrical stimulation. The constructs were placed in fresh medium and oriented with platinum electrodes aligned colinear to the axis of myotubes. A square wave stimulus was applied at 1 V mm^−1^ with 4 ms pulse duration at stimulation frequencies of 1 and 5 Hz (Figure 7C). Myotube contractions were recorded via live imaging, and the displacement amplitude was quantified (see Videos S4 and S5, Supporting Information showing contractions at 1 and 5 Hz, respectively). The constructs responded synchronously to the applied electrical stimulus. At 1 Hz, the average displacement amplitude was ≈4 µm, while at 5 Hz, the amplitude increased to ≈7 µm, consistent with the onset of tetanic contractions. These results confirm that Pax7‐nGFP myoblasts, when encapsulated in the optimized resin formulations and stored for up to one week and printed in microgravity, retain their capacity to form structurally mature and functionally contractile muscle tissue constructs. Of note, the values demonstrated in Figure 7C are for one representative construct from the CoolResin_4C and CryoResin_4C formulations. Consequently, statistical comparison between the groups and frequency was not possible, which is a limitation given by the complex nature of the experiments in our present work. Due to the limited availability of the constructs, a comparison of µG‐printed with On‐Ground‐printed groups in terms of the presence of sarcomere structures was also not performed. Analysis with higher sample sizes for constructs printed on microgravity or on‐ground will be an important consideration for our future work.

## 3 Discussion

Biofabrication of engineered tissues and grafts in microgravity environments can help sustain long‐term space missions and provide insights into disease mechanisms in space.^[^ ^8^, ^13^, ^47^ ^]^ In this work, we used G‐FLight as gravity‐independent approach for the rapid biofabrication of anisotropic muscle tissues. We have used the term “gravity‐independent” in context with the similarity of the microgravity‐printed tissues to those fabricated on‐ground. This is largely enabled due to the rapid (i.e., within seconds) fabrication using the G‐FLight system and the GelMA‐based resin formulations, where the cells were confined in a viscous hydrogel material (especially within the formulations printed at 4 °C). The characteristics of the matured muscle tissues (e.g., myotube diameter, fusion index, and sarcomere spacing) printed using the G‐FLight system are comparable to existing approaches using casting,^[^ ^45^, ^48^, ^49^ ^]^ micropatterning,^[^ ^50^, ^51^ ^]^ and the native mouse muscle.^[^ ^52^ ^]^ These attributes support the potential use of G‐FLight system for the fabrication of muscle constructs in space, with notable use in disease modeling,^[^ ^53^ ^]^ or as regenerative grafts.^[^ ^54^ ^]^ For instance, one of the unfavorable attributes of space travel is the rapid atrophy of skeletal muscle.^[^ ^55^, ^56^ ^]^ Here, the printed muscle constructs can be used as a platform to screen for means to maintain or regenerate muscle mass or prevent its loss in microgravity (small molecules, exercise regimen, etc.),^[^ ^57^ ^]^ as a potential next exploratory step. Other growing research avenues, including biohybrid muscle actuators,^[^ ^58^ ^]^ or cultivated meat,^[^ ^59^ ^]^ can potentially also be explored with this method. Specific to muscle tissue engineering, our future studies will assess changes in gene expression and downstream effects after long‐term storage, and also incorporate high‐resolution imaging of actin microfilament organization and quantification of myotube alignment relative to the contraction axis to elucidate structure–function coupling.^[^ ^60^, ^61^ ^]^ In addition to sarcomeric striation periodicity, contraction vector mapping will also be used to assess axial consistency and functional maturation.^[^ ^45^, ^61^ ^]^ While we only encapsulated cells at 10^6^ cells mL^−1^, future work will also investigate encapsulation of cells at higher densities to enable higher contraction forces and higher myotube densities within the constructs. Here, refractive index matching by the addition of biocompatible contrast agents (e.g., Iodixanol) may be necessary to minimize light scattering at high cell densities and allow prevalence of microfilaments throughout the constructs.^[^ ^62^, ^63^, ^64^ ^]^

While this study focused on Pax7‐nGFP primary mouse myoblasts, which are known for their high regenerative capacity and resilience to environmental stress,^[^ ^65^, ^66^ ^]^ it will be important to test whether these resin formulations and the G‐FLight system are broadly compatible with other, potentially more sensitive, human cells (e.g., mesenchymal stem cells (MSCs), or induced pluripotent stem cell‐derived cells, etc.).^[^ ^67^, ^68^ ^]^ As pilot on‐ground experiments, we performed additional experiments using human MSCs encapsulated (at 10^6^ cells mL^−1^) within each resin formulation (ControlResin, Coolresin and CryoResin). For simplicity and ease of comparison, we only chose 4 °C as the printing temperature. Here, the MSCs demonstrated high viability at days 1 and 7 of culture (Figure S7, Supporting Information), indicative of the promising potential of the resins in enabling survival of different cell types. Our future studies would investigate the effects of the resins and processing steps on the differentiation of the cells and functional maturation of the tissues derived thereof. The printing approach and materials could potentially also be deployed towards other tissues such as cartilage,^[^ ^19^, ^69^ ^]^ tendon,^[^ ^44^ ^]^ neurons,^[^ ^18^ ^]^ or cardiovascular,^[^ ^70^ ^]^ which have been previously printed on‐ground using FLight biofabrication.

In this work, the use of physical masks to shape the light patterns allowed miniaturization of the light engine in the G‐FLight system, to be able to conform to the permissible dimensions of the airplane rack. However, the use of physical masks limits the flexibility of the shapes that can be fabricated; for instance, hollow tubular shapes are not possible with the current system since the inner circle of the tube will be a free‐floating structure within the 3D printed mask. If a larger size of the printing system may be permissible in the future, the use of a DMD for light shaping could obviate this limitation.^[^ ^17^, ^19^ ^]^ Importantly, we have also compared the resolution we could obtain with the physical masks in the G‐FLight system to that using the desktop FLight device from our previous work,^[^ ^17^, ^41^ ^]^ where a DMD was used for image control (Figure S8A, Supporting Information). For these studies, the light dose (in mJ cm^−2^) was kept the same as that optimized for each formulation used in Figure 4A, enabling direct comparison for each material formulation. The minimum feature sizes achieved with the desktop FLight device were smaller than those obtained with the G‐FLight printer (Figure S8B,C, Supporting Information). This can be attributed to the smaller pixel pitch of its DMD (7.56 µm) compared to the feature size of the 3D‐printed photomasks, which allowed projection of finer image features into the resin‐filled cuvette.

Another limitation of FLight printing is that it is currently limited to 2.5D prints, where the cross‐section is constant throughout the fabricated structures.^[^ ^20^, ^28^ ^]^ Here, other deep vat printing approaches such as tomographic printing,^[^ ^71^, ^72^ ^]^ or light sheet‐based printing (e.g., Xolography),^[^ ^73^, ^74^, ^75^ ^]^ can be deployed using the same bioresins which we have shown in this work, to produce complex 3D constructs.^[^ ^76^, ^77^, ^78^ ^]^ Recently, the projection of multi‐wavelength light patterns has allowed selective photocrosslinking or photodegradation within multimaterial bioinks.^[^ ^28^, ^79^, ^80^ ^]^ These strategies can enable the on‐demand fabrication of complex multi‐cellular tissues, reinforcing the rationale for conducting tissue biofabrication in space rather than transporting prefabricated tissues on space missions. Even with the limited flexibility offered by physical masks, one can deploy the resin swapping technique similar to our prior work,^[^ ^64^, ^81^ ^]^ to be able to achieve multi‐material prints using G‐Flight system. As a proof‐of‐concept demonstration, we created multi‐cellular constructs, where human umbilical vein endothelial cell (HUVEC)‐laden resin (we used CoolResin for this demonstration) formed channels within a hexagonal‐shaped construct made of Pax7‐nGFP myoblast‐laden resin (Figure S9, Supporting Information). Notably, performing the resin swapping activities in microgravity conditions aboard a spacecraft would be operationally challenging. Here, hybrid bioprinting approaches, such as combining light projection techniques with extrusion bioprinting,^[^ ^76^, ^79^ ^]^ could allow compartmentalization of different cell types or materials within the cuvettes, enabling multimaterial printing with a single light projection in the G‐Flight system. It remains to be seen, preferably under perfusion culture systems,^[^ ^77^ ^]^ how the microgravity conditions affect the growth, alignment, and differentiation of different cell types within these constructs.

During the microgravity campaign, when onboard the parabolic flight, the thermo‐reversibly gelled state of the resins stored in the on‐board refrigerator (4 °C) allowed maintenance of shape fidelity of the printed constructs within the cuvettes in the different cycles of the microgravity flight (0, 1 or 1.8 g phases). However, when going aboard typical spacecrafts which exhibit 3–4 g of forces during take‐off and re‐entry,^[^ ^78^ ^]^ as well as for long‐term space missions (such as aboard the ISS), storing the resin formulations at 4 °C may not be sufficient to allow maintenance of cell viability and shape fidelity.^[^ ^82^, ^83^ ^]^ In this context, the CryoResin formulation presents a promising platform for taking cellular resins aboard spacecrafts and for long‐term missions (several months to years). A solid state of the resin at −80 °C will enable resilience to high G‐forces, while the resin thawing, refrigeration (for 4 °C printing temperature), and printing activities could be performed in microgravity conditions. The CryoResin formulations could be supplemented with broadly used cryopreservation techniques and formulations,^[^ ^84^, ^85^ ^]^ potentially enabling wider application across different cell types, biomaterial formulations, and off‐the‐shelf biofabrication workflows.^[^ ^64^, ^65^ ^]^ Importantly, the resin constituents from this work (i.e., MH, HTS and DMSO) can possibly also be transferred to other biomaterial formulations, such as those based on collagen,^[^ ^41^, ^86^ ^]^ hyaluronic acid,^[^ ^87^ ^]^ silk,^[^ ^88^, ^89^ ^]^ decellularized matrix,^[^ ^90^ ^]^ or fibrinogen,^[^ ^91^ ^]^ etc., which have been widely used in tissue engineering and regenerative medicine. These formulations have been used as non‐modified (e.g., using native tyrosine crosslinking) or functionalized (e.g., methacrylated, or norbornene and thiol‐functionalized) with FLight or volumetric printing,^[^ ^19^, ^41^, ^88^, ^92^ ^]^ and can expand the range of tissues which can be created.

While not in the scope of the present study, transferring of cryopreserved constructs from −80 °C storage into liquid N_2_ (−196 °C), with subsequent successful retrieval and printing, would support the feasibility of using such formulations in long‐duration space missions. Accordingly, we have performed additional pilot experiments (Figure S10, Supporting Information) for the storage of CryoResin formulations containing Pax7‐nGFP primary mouse myoblasts in liquid N_2_ after cryofreezing. After thawing and printing using the same procedures as those for the formulations stored at −80 °C, the constructs were washed and cultured. The cells in the resins stored in liquid N_2_ demonstrated similar viability at Days 1 and 7 as the resins stored at −80 °C (Figure S10, Supporting Information), which demonstrates the promising potential of these resins for use in long‐term space missions.

While longer duration space missions may require cryopreservation of the resin formulations, shorter duration (< 2 weeks) could suffice with CoolResin formulations, which are simpler in composition and inherently more biocompatible due to the absence of DMSO. With these formulations, printing at physiological temperature (37 °C) may be beneficial for cell survival and would therefore be a future scope of exploration, if the printing window permits the longer crosslinking duration needed at higher temperatures. Importantly, longer printing durations can also affect tissue maturation, as evidenced by the CryoResin_37C formulations, which did not demonstrate uniaxial myotube alignment due to a reduced porosity emerging from the free radical diffusion (the prints required longer light exposure compared to other formulations) and subsequent non‐specific crosslinking. Here, one could deploy resin formulations leveraging rapid photoclick chemistries (such as thiol‐ene click reactions),^[^ ^64^, ^81^ ^]^ to allow faster prints at physiological printing temperature.

When using light‐based biofabrication systems in space, as a standalone or hybrid approach, one must consider the steps for washing away the uncrosslinked resin and culturing the tissues under perfusion. In the future, we will investigate the design of customized print containers, which could allow easy coupling to perfusion systems for the removal of the uncrosslinked resin, followed by media flow to facilitate tissue maturation.^[^ ^7^ ^]^ Such perfusion‐based culture systems can also be tested under simulated microgravity, i.e., tissues can be fabricated in parabolic flights followed by maturation under perfusion in simulated microgravity (e.g., in RPM machines).^[^ ^93^ ^]^ However, there are differences in the cellular adhesion, mechanosensitive gene expression, and mitochondrial function in the cells when comparing simulated microgravity to real microgravity, such as in space flights.^[^ ^82^, ^94^ ^]^ Therefore, biofabrication and long‐term culture within real microgravity, such as aboard the ISS,^[^ ^12^, ^83^ ^]^ will also be investigated in our future work.

## 4 Conclusion

We have presented G‐FLight printing as an effective tool for the rapid gravity‐independent fabrication of aligned tissues, focusing on muscle tissue as an application. The printer featured a robust and compact form‐factor capable of printing aligned microfilamented tissue constructs within seconds. We optimized specific resin formulations, which allowed storage together with encapsulated cells under refrigeration or cryopreservation conditions for at least one week. The printed constructs from optimized resin formulations exhibited increased cell survival (>80% viability) compared to the controls (< 50% viability) after a week in culture. In the matured tissue constructs which were printed in microgravity, we observed higher myotube density and fusion index compared to controls and similar myotube characteristics to the constructs which were printed on‐ground. We also observed sarcomere structures in the tissue constructs made from optimized formulations, with spontaneous myotube contractility and synchronization to external stimulus. By demonstrating gravity‐independent FLight printing together with cell‐laden resin formulations, which allow long‐term storage, this study can pave the way for new light‐based approaches for tissue biofabrication in space.

## 5 Experimental Section

### Assembly of the G‐FLight Printer

The printer components were mounted onto an optical aluminum breadboard (30 × 45 cm, MB3045/M) containing M6 taps spaced‐out by 25 mm. These taps were used to mount all the printer components including the cover to allow a robust assembly. The refrigeration unit for the storage of the resins at 4 °C during the parabolic maneuvers was a commercially available insulin storage unit box utilizing a Peltier cooling system, and the heating unit consisted of a custom 3D printed Polyethylene terephthalate glycol (PETG) enclosure with a 3D printed cap and an aluminum jacket wrapped around for even heat distribution. The heat was generated by wrapping a mini flask warmer (Thermup Go, Lionel Care), which was set to 41 °C, allowing maintenance of 37 °C inside the heating unit to liquefy the resins before printing (only for the formulations that were printed in a heated state). The other printer components, including the mask holder, masks, printing enclosure, and the cover of the printer, were also custom 3D printed using PETG and mounted onto the optical breadboard.

As for the optical and electrical components, a supplementary figure has been provided detailing the components and their connections (see Figure S11, Supporting Information). The laser module (LDM‐405, LaserTack GmbH) was purchased as a collimated source with anamorphic pairs mounted at the output to generate a square beam profile (4 × 4 mm) at 300 mW. A top hat diffuser (ED1‐S20‐MD, Thorlabs) was placed along the laser light path to homogenize the beam (i.e., remove the Gaussian intensity distribution) at the output of the laser. A plano‐convex lens (f = 75 mm, Thorlabs) was then used to collimate the light beam, which was subsequently shaped using the masks inserted into the printing chamber. The optical components were covered by an aluminium enclosure to prevent light leakage as per the safety norms dictated by NoveSpace. Additional laser indication lights were connected to the laser controller to turn ON when the laser was activated. In addition, a reed switch was mounted onto the printing closure (where the masks and cuvettes were inserted) and was interlocked with the laser controller. A magnet was attached to the lid of the printing enclosure, which, upon closing, activated the laser. The laser controller received its analog signals through a Raspberry Pi 4.0, which in‐turn received user input for the light intensity and the print duration through a graphical user interface displayed on a touch screen. All the components were powered by a single DC power supply unit (12 V, 50 W, LRS 50‐12, Mean Well), which was connected to the main supply. The components running on 12 V (Laser module and refrigeration unit) were directly connected to the power supply (with appropriate fuses), while the components requiring 5 V supply (Raspberry Pi and heating unit) were interfaced with a 12–5 V converter (LM2596, Purecrea, Bastelgarage) between the component and the power supply.

### Photoresin Constitution

Gelatin methacryloyl (GelMA) with a high degree of functionalization (DoF) was synthesized following our established protocol. Briefly, Type A gelatin (G2500, Sigma) was dissolved at 10% (w/v) in 0.25 m carbonate–bicarbonate buffer (pH 9) at 50 °C under constant stirring. Methacrylic anhydride (MAA, 276685‐500, Sigma) was added in five equal portions over the course of 2 h (one addition every 30 min), maintaining vigorous stirring and constant pH (9.0) throughout the reaction. A total MAA‐to‐gelatin ratio of 0.4 mL per gram of gelatin was used. One hour after the final addition, the reaction was terminated by two‐fold dilution with deionized water and pH adjustment to 7.4. The solution was centrifuged to remove unreacted MAA and then dialyzed (12–14 kDa MWCO, 3110, Merck) against Milli‐Q water for 5 days with frequent water changes. The purified solution was sterile filtered (0.22 µm) and lyophilized. GelMA was stored at −20 °C until use.

To quantify the DoF, both unmodified gelatin and GelMA were dissolved at 1% (w/v) in deuterium oxide and analyzed by ^1^H NMR (Bruker Ultrashield 400 MHz, 64 scans), see Figure S12 (Supporting Information). Spectra were normalized to the aromatic proton peaks of phenylalanine (7.1–7.5 ppm), which remain unaltered during functionalization. The ε‐methylene protons of lysine (2.95–3.05 ppm) were integrated and compared between gelatin and GelMA to calculate the DoF using Equation (1) and found to be 99%.

(1) $$DoF\;\left[\%\right]=\left(1-\frac{\int \mathrm{lysine}\;\;\mathrm{methylene}\;\mathrm{signals}\;\left(GelMA\right)}{\int \mathrm{lysine}\;\mathrm{methylene}\;\mathrm{signals}\;\left(gelatin\right)}\right)\cdot 100$$

The ControlResin formulations were created by simply dissolving lyophilized GelMA at 5% w/v in PBS and allowing dissolution of the resin for 30–45 min at 50 °C. The resin was allowed to cool down to 37 °C followed by adding LAP (Lithium phenyl‐2,4,6‐trimethylbenzoylphosphinate) as the photoinitiator. For constituting the CoolResin or CryoResin formulations, dissolution solutions were first prepared by mixing HTS (for CoolResin) or a combination of HTS, MH, and DMSO (for CryoResin) at the desired concentrations (Figure 1B), first in PBS and later adding GelMA at 5% w/v and LAP at 0.1% w/v (same dissolution procedure as the ControlResin formulations). The resins were freshly prepared the day before cell encapsulation or any in vitro evaluation and were stored at 4 °C until used. Before usage, the resins were liquefied at 37 °C for 5 min followed by further processing (i.e., encapsulation of cells or addition into cuvettes).

### Cell Culture, Bioresin Constitution, and Tissue Maturation

The Pax7‐nGFP mouse primary myoblasts and related culture methods were kindly supplied by Prof. Ori Bar‐Nur. To grow and expand myoblasts in 2D conditions, the culture flasks were pre‐coated with a coating solution, consisting of 1 mL of Matrigel (Corning, 356237) in 24 mL of low‐glucose DMEM (Thermo Fisher Scientific, 31885023) and 1% v/v Penicillin–Streptomycin (Thermo Fisher Scientific, 15140122). After adding the coating solution, the culture flasks were incubated in the fridge for 7 min, the coating solution was collected, and flasks incubated at 37 °C for a minimum of 1 h before use. A myoblast expansion medium comprised of Dulbecco's Modified Eagle Medium (DMEM, Thermo Fisher Scientific, 41966029) and F‐10 Medium (Thermo Fisher Scientific, 22390025) in equal parts, which was further supplemented with 10% v/v horse serum (Thermo Fisher Scientific, 16050122), 20% v/v fetal bovine serum (FBS, Thermo Fisher Scientific, A5256701), 1% v/v Penicillin‐Streptomycin (ThermoFisher Scientific, 15140122), and 10 ng mL^−1^ recombinant FGF2 (Bio‐Techne, 233‐FB‐500). The differentiation medium comprised of 1% v/v Penicillin‐Streptomycin (ThermoFisher Scientific, 15140122), 1% v/v Insulin‐Transferrin‐Selenium (Gibco, 41400‐045), and 2% v/v horse serum (Thermo Fisher Scientific, 16050–122) added to DMEM (Thermo Fisher Scientific, 31966‐021).

Human umbilical vein endothelial cells (HUVECs; Lonza) were cultured under standard conditions (37 °C, 5% CO_2_) in Endothelial Cell Growth Medium‐2 (EGM‐2 BulletKit, Lonza). Human mesenchymal stem cells (MSC; cell line; Lonza) were cultured under standard conditions (37 °C, 5% CO_2_) in MEMα (Thermo Fisher Scientific, 22561‐054) supplemented with 10% FBS (Thermo Fisher Scientific, A5256701), 10 µg mL^−1^ Gentamycin (Thermo Fisher Scientific, 15710049) and 5 ng mL^−1^ FGF2 (Thermo Fisher Scientific, 100–18B). The cell culture for the hydrogel constructs was performed for up to a week in the same media formulation as for the normal cell culture.

Upon reaching 80% confluency, the cells were harvested using Trypsin‐EDTA 0.25% (ThermoFisher, 25200056) treatment, centrifuged at 500 g for 4 min, and suspended in the different resin formulations at 10^6^ cells mL^−1^. After printing (on‐ground or in microgravity), the hydrogel constructs with Pax7 cells were first cultured in expansion medium for 7 days (37 °C with 5% CO_2_), with the medium refreshed every 2 days. The tissue constructs were then incubated in differentiation medium for 10 days, where the media was replaced every 2 days, and once spontaneous muscle contractions were detected, the medium was changed daily.

### Photoresponse Characterization of the Resin Formulations

Photorheology experiments were conducted as per our previous study,^[^ ^95^ ^]^ using a rheometer (MCR 302e, Anton Paar) fitted with a 20 mm serrated plate geometry and a glass base. A UV lamp (Omnicure Series 1000, Lumen Dynamics) was used along with 400‐500 nm bandpass filters (Thorlabs). To prevent sample drying, a moist tissue paper was placed in the chamber during testing. Oscillatory measurements were performed in triplicates at 37 °C, using 152 µL of photoresin, with a 2% shear rate, 1 Hz frequency, a 200 µm gap, a 10 s acquisition intervalat an intensity of 2 mW cm^−2^.

### Mechanical Testing of the Crosslinked Constructs

Unconfined compression testing was conducted using a Texture Analyzer (TA.XTplus, Stable Micro Systems) fitted with a 0.5 kg load cell. After positioning the sample between two compression plates, a pre‐load of 0.3 grams was applied to ensure complete contact with the samples. Following a relaxation period, the samples were compressed to 15% strain at a rate of 0.01 mm s^−1^. The loading and unloading curves were recorded, and the compressive modulus was determined by performing a linear fit on the stress‐strain curve as per our previous work.^[^ ^96^ ^]^

### Light Sheet Imaging of the Printed Constructs

Light sheet imaging of the construct‐laden cuvettes was performed as per the methods described in our previous work.^[^ ^81^ ^]^ Briefly, acryloxyethyl thiocarbamoyl Rhodamine B (Rhod‐Acr) stock in DMSO (at 10 mg mL^−1^) was added to the resin formulation at 1 µL mL^−1^ to enable fluorescence imaging. For facilitating imaging, the constructs were printed in 4 mm path length glass cuvettes (Thorlabs), which allowed the constructs to stick to the cuvettes.^[^ ^81^ ^]^ The constructs were washed 4 times with warm PBS at 37 °C to remove any uncrosslinked resin. A light sheet microscope with axial scanning capability (MesoSPIM, V4) was employed to capture images of fluorescently labeled samples. The cuvettes with the constructs were placed on the MesoSPIM microscope stage, and imaging was conducted using a macro‐zoom system (Olympus MVX‐10) and a 2x air objective lens (Olympus) with adjustable zoom functionality. For each imaging session, voltage adjustments were made using an electrically tunable lens (ETL). The step size for imaging ranged between 10 and 50 µm.

### Microfilament Imaging and Size Assessments

The rhodamine‐labeled resins (method for fluorescent labeling is described in the previous subsection) were imaged using a confocal microscope. To quantify the 3D porosity of the hydrogels, confocal image stacks were converted into Imaris‐compatible file formats using the Imaris File Converter software (Oxford Instruments, Ver. 10.2.0), and subsequently imported into Imaris (Oxford Instruments, Ver. 10.2.0) for surface reconstruction. Filament surfaces were rendered using the software's standard surface detail settings and default intensity thresholds, unless stated otherwise. This analysis was performed for three different hydrogel matrices, and the filament volume obtained from surface reconstructions was used to compute 3D porosity.

For microfilament diameter characterization, the same confocal image stacks were processed using Fiji (ImageJ, Ver 1.54m). Z‐projections of the top, middle, and bottom regions of each stack were generated, and filament diameters were determined by measuring the full width at half maximum (FWHM) of the resulting intensity profile.

### Live/Dead Assessments of the Muscle Constructs

For viability assessments on Days 1 and 7, the cells in the constructs were stained with Hoechst 33342 (Invitrogen, 1:1000), propidium iodide (PI, Fluka, 1:500), and CalceinAM (Invitrogen, 1:2000). For assessment, a confocal laser scanning microscope (Fluoview 3000, Olympus) was utilized to capture 100 µm z‐stacks with a 3 µm step interval (*n* = 3). Image stacks were converted to Imaris‐compatible format, imported into Imaris, and analyzed using the spot detection function to identify and count total nuclei and PI⁺ nuclei. The percentage of PI⁺ was calculated as the number of PI⁺ cells divided by the total number of nuclei. The percentage of Calcein‐AM⁺ cells was calculated as: 100 – (% PI⁺ cells).

### Analysis of Myotube Alignment

Confocal images of matured constructs were analyzed in Fiji (ImageJ) using the OrientationJ plugin (http://bigwww.epfl.ch/demo/orientationj). Orientation distributions were computed with the Distribution tool after estimating local orientation via the structure tensor using a Gaussian gradient and a tensor window of 20 px (>2 × myotube diameter). For each condition, three independent image stacks were analyzed. For comparison across samples, each orientation histogram was centred on its dominant alignment peak and wrapped to the range [−90°, 90°]. The percentage of aligned myotubes was defined as the fraction of signal oriented within ±10° of the dominant orientation peak.

### Immunohistochemistry and Analysis of the Muscle Constructs

The constructs were fixed using 4% v/v paraformaldehyde for 30 minutes at room temperature, followed by washing with PBS. Before staining, the cells in constructs were permeabilized by treating the constructs with 0.2% v/v Triton X‐100 in PBS for 15 min, followed by three washes in PBS. Blocking was performed with 5% w/v bovine serum albumin (BSA) in PBS for 1 h. The constructs were subsequently incubated with either a primary anti‐myosin heavy chain antibody (MF‐20, DSHB, anti‐mouse, 1:20 in PBS + 1% v/v BSA) or an anti‐alpha‐actinin (sarcomeric) antibody (A7811, Sigma, anti‐mouse, 1:500 in PBS + 1% v/v BSA), or anti‐ki67 antibody (550609, BD BioSciences, anti‐mouse 1:200 in PBS + 1% v/v BSA) together with anti‐MyoD antibody (PA5‐23078, Invitrogen, anti‐rabbit, 1:100 in PBS + 1% v/v BSA) for 24 h under constant agitation. After three additional 15 min PBS washes, the constructs were incubated with appropriate secondary antibodies: Goat anti‐mouse (AlexaFluor488, Invitrogen, 1:500 in PBS + 1% v/v BSA) and/or Goal anti‐rabbit (AlexaFluor647, A‐21244, Invitrogen, 1:500 in PBS + 1% v/v BSA). Hoechst 33342 (H3570, Invitrogen, 1:1000 in PBS + 1% v/v BSA), and phalloidin‐tetramethylrhodamine B isothiocyanate (P1951, Invitrogen, 1:1000 in PBS + 1% v/v BSA) at 4 °C for 2 h. Finally, the samples were washed three times in PBS before being imaged using confocal microscopy (Fluoview 3000, Olympus).

### Assessment of Contractile Response of the Muscle Constructs

For stimulation, the matured tissue constructs for the CoolResin and CryoResin formulations were transferred into 12 well plates filled with 1 mL of differentiation media. Separately, on the lid of the 12 well plates, 1 mm diameter holes were drilled and 1 mm diameter platinum electrodes inserted (17.5 mm spaced‐out). The electrodes received electrical stimulus through an external power source, which was interfaced to an Arduino via a TIP120 transistor. The voltage on the power source was set to achieve a 1 V mm^−1^ voltage gradient parallel to the fiber orientation. The Arduino code was set to stimulate the muscle constructs using 4 ms pulses at 1 or 5 Hz. The setup was then placed atop a brightfield microscope (M5000, EVOS), and the video recordings were performed at 60 fps using an external camera. The recordings were taken of both the spontaneous and electrically responsive contractions of the muscle constructs. The plots for the displacement of the myotubes were determined using a custom python script analyzing the video frames based on our existing study.^[^ ^41^ ^]^

### Desktop FLight Printing for Comparing Image Resolution With the G‐FLight System

The desktop FLight printing concept was based on our prior work,^[^ ^41^, ^64^ ^]^ where a 405 nm fiber coupled laser (FL‐405‐1200, Lasertack GmbH) projected a collimated speckled light beam (collimation achieved using a telescopic lens set) into a digital micromirror array device (DLPLCR65EVM, Texas Instruments) with a 7.56 µm pixel pitch. The reflected image from the DMD was captured using another set of telescopic lenses with an iris in‐between (to clean‐up the fringe patterns in the reflected image) and projected as a collimated image pattern into a resin‐filled cuvette. The light dose achieved at the cuvette using the desktop FLight printer at 100% power was ≈64 mW cm^−2^, which was higher than the G‐Flight setup (34 mW cm^−2^). Accordingly, the light exposure duration was calibrated to achieve consistent light doses with the G‐FLight device and enable comparison between the two different printing systems.

### Statistical Analysis

Statistical analysis was carried out in GraphPad Prism (v. 10.2.3) using One‐way ANOVA followed by Tukey's post‐hoc analysis to compare groups. A significance level of 0.05 was applied. Statistical significance is indicated as follows: *p* < 0.05 (*), *p* < 0.01 (**), *p* < 0.001 (***), and *p* < 0.001 (****); “ns” denotes no significant difference.

## Conflict of Interest

The authors declare no conflict of interest.

## Supporting information



Supporting Information

Supplemental Video 1

Supplemental Video 2

Supplemental Video 3

Supplemental Video 4

Supplemental Video 5

## Data Availability

The data that support the findings of this study are openly available in ETH Research Collection at https://doi.org/10.3929/ethz‐b‐000739779, reference number 739779.
