# Supplementary material for: Prolonged Cell Encapsulation and Gravity‐independent Filamented Light Biofabrication of Muscle Constructs
Source: Adv Sci (Weinh). 2025 Sep 23;12(45):e12727. doi: 10.1002/advs.202512727 (PMC12677601; doi:10.1002/advs.202512727)
Supplement: Supplementary file 1 — Supporting Information [file ADVS-12-e12727-s006.docx]

**Prolonged cell encapsulation and gravity-independent filamented light biofabrication of muscle constructs**

Michael Winkelbauer^1^, Jakub Janiak^1^, Johannes Windisch^2^, Hao Liu^1^, Maria Bulatova^1^, Max Von Witzleben^2^, Hugo Oliveira^3^, Sophie Dani^2^, Richard Frank Richter^2^, Nicolas L’Heureux^3^, Ori Bar-Nur^4^, Michael Gelinsky^2^, Marcy Zenobi-Wong^1^, Parth Chansoria^1*^

^1^ Institute for Biomechanics, Department of Health Sciences and Technology, ETH Zürich, Switzerland

^2^ Center for Translational Bone, Joint and Soft Tissue Research, University Hospital and Faculty of Medicine, TU Dresden, Fetscherstr. 74, 01307 Dresden, Germany

^3^ Univ. Bordeaux, Tissue Bioengineering INSERM U1026, F-33000 Bordeaux, France

^4^ Laboratory of Regenerative and Muscle Biology, Department of Health Sciences and Technology, ETH Zurich, Schwerzenbach, Switzerland

*Correspondence: [parth.chansoria@hest.ethz.ch](mailto:parth.chansoria@hest.ethz.ch)

This file contains **Supplementary** **Figures S1 to S12**


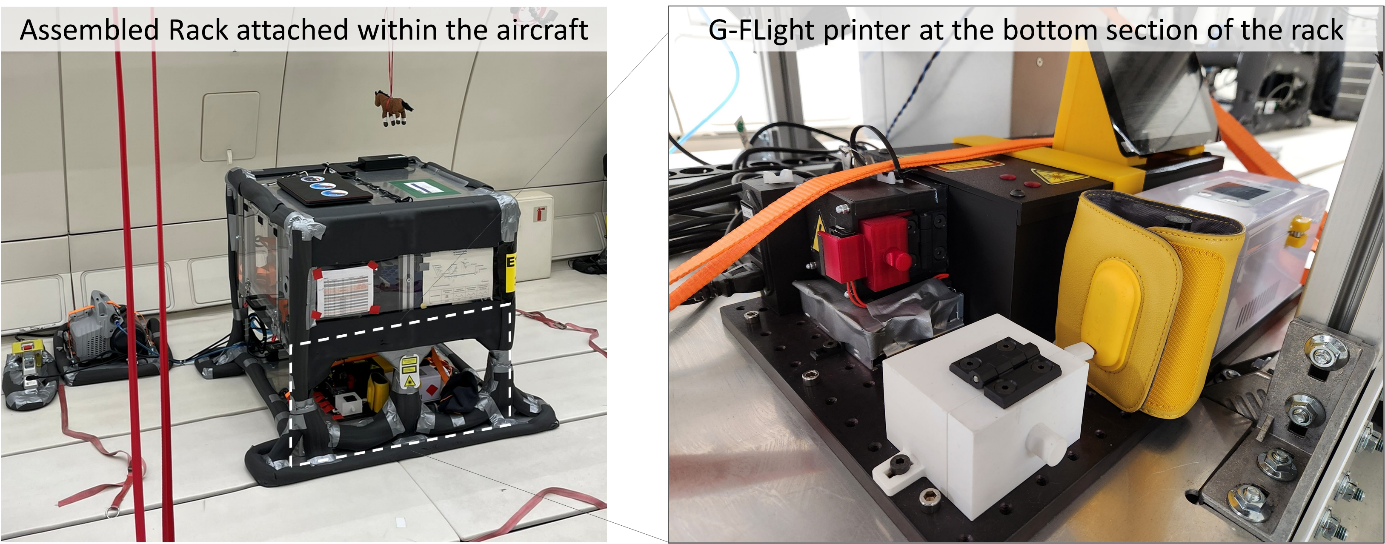


**Figure S1.** Photograph of the rack installed within the aircraft (left) and photograph of the printer attached within the rack (right).

**Figure S2.** Compressive modulus of the printed formulations under different exposure duration using the same light engine (34 mW/cm^2^).


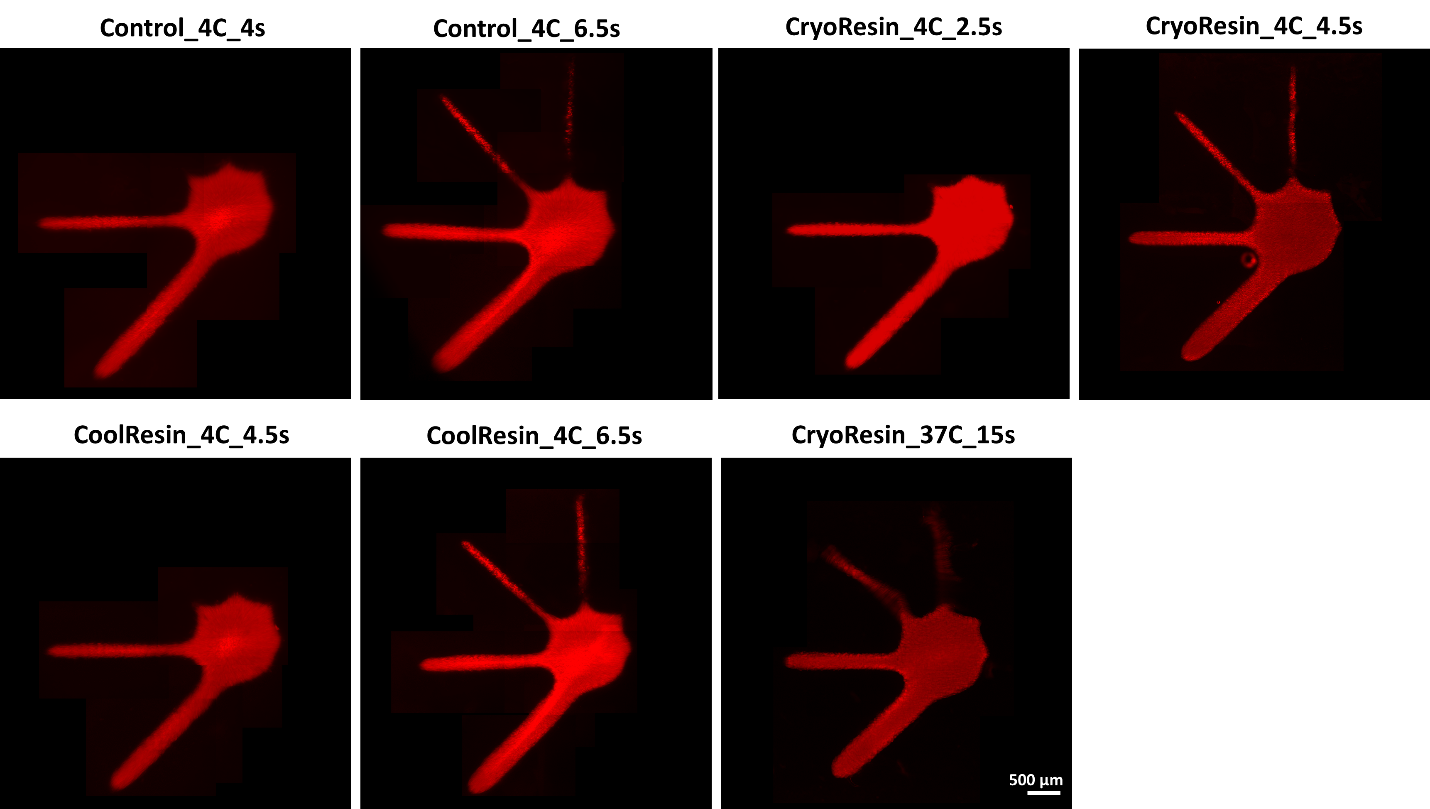


**Figure S3.** Resolution images of the selected resin formulations printed using the G-FLight system. Of note, the longer exposure durations allow emergence of thinner features within the constructs.


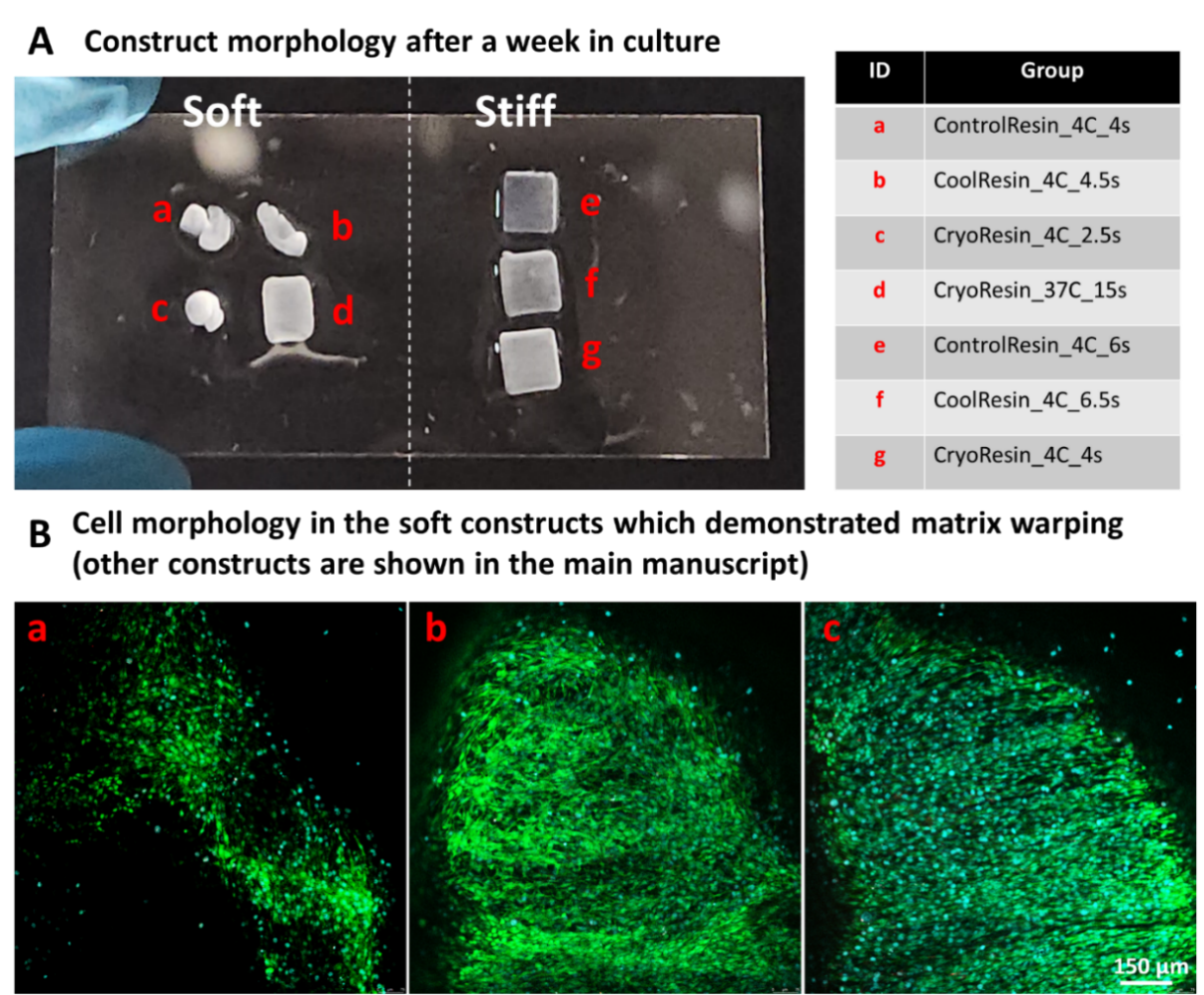


**Figure S4. A.** The printed constructs (image on the left) and their compositions (table on the right) after a week in culture. **B.** The morphology of the soft constructs (The resin compositions for the groups a, b and c can be found in the table in A), which demonstrates significant warping within the ControlResin and CoolResin formulations.


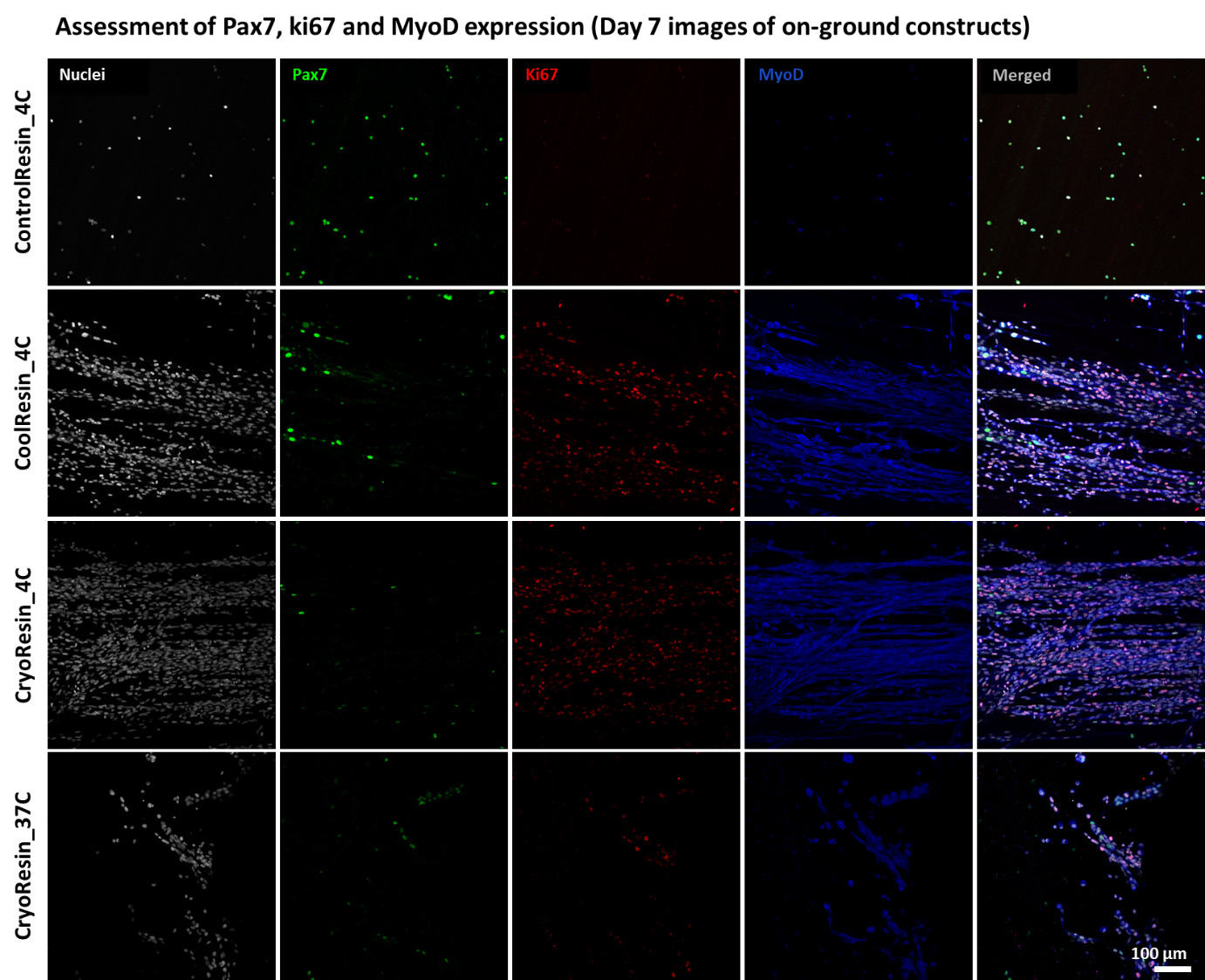


**Figure S5.** Staining for nuclei (grey), Pax7+ (green), ki67+ (red) and MyoD+ (blue) signals within the On-Ground printed constructs after a week in culture.


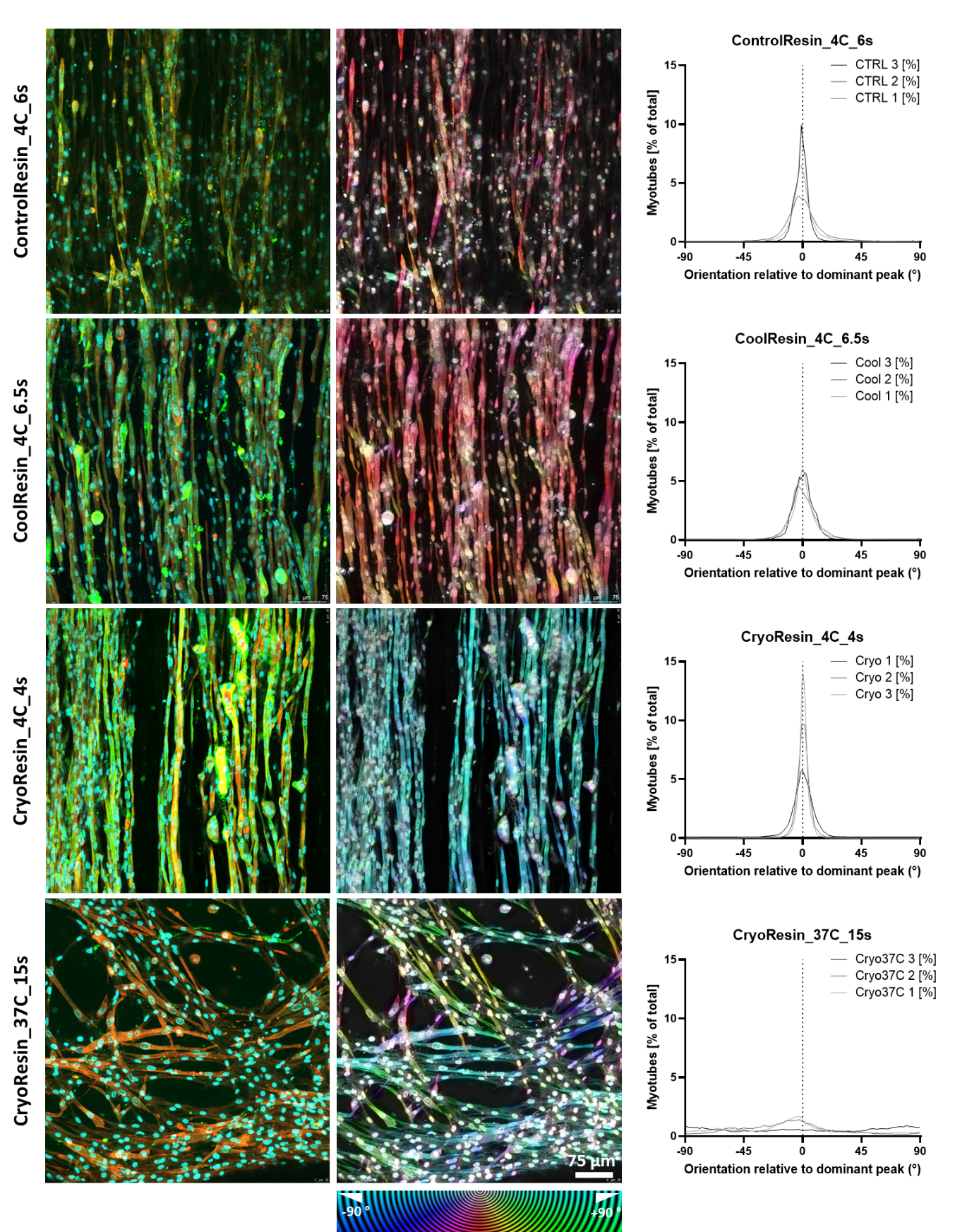


**Figure S6.** Quantification of f-actin alignment across different regions. Coloured boxes indicate the summed proportion of aligned actin filaments within ±10° of the dominant orientation peak.

**
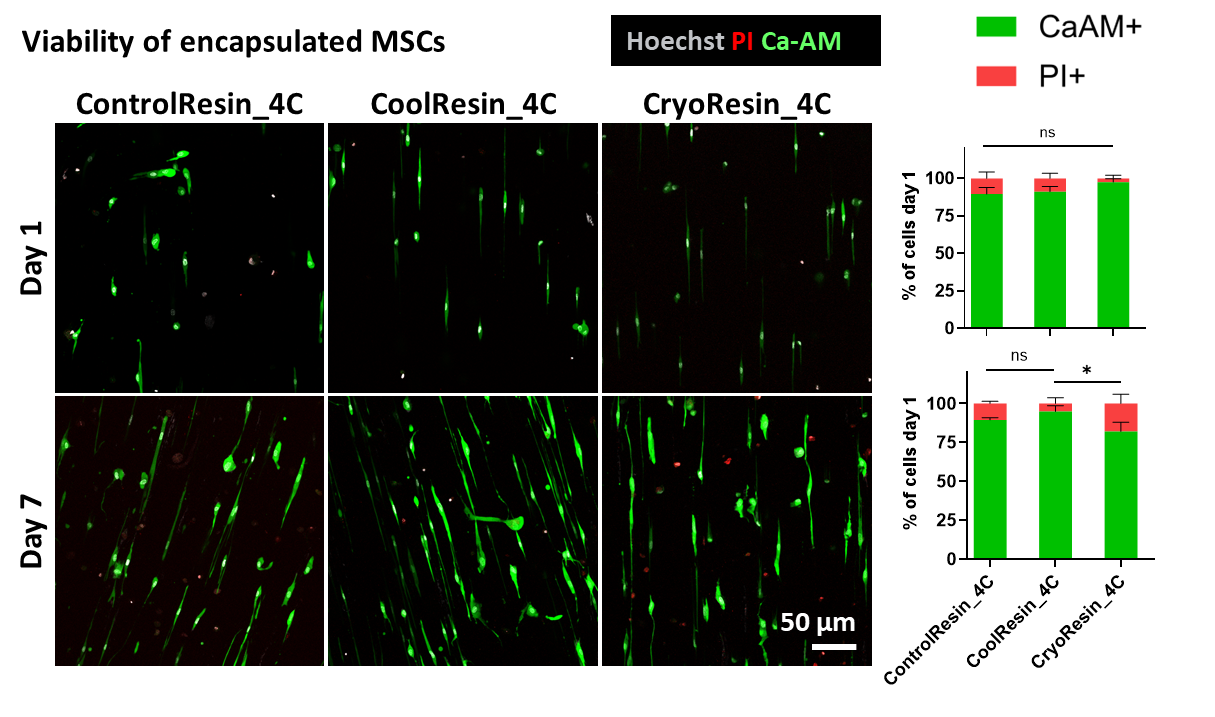
**

**Figure S7.** Viability of encapsulated-MSCs within the different resin formulations. The cell viability is high across a week in culture, even within ControlResin formulations, indicating the robustness of these cells in withstanding prolonged storage in refrigeration condition (4°C). These tests also indicate the cytocompatibility of the CoolResin and CryoResin formulations with other types of cells.


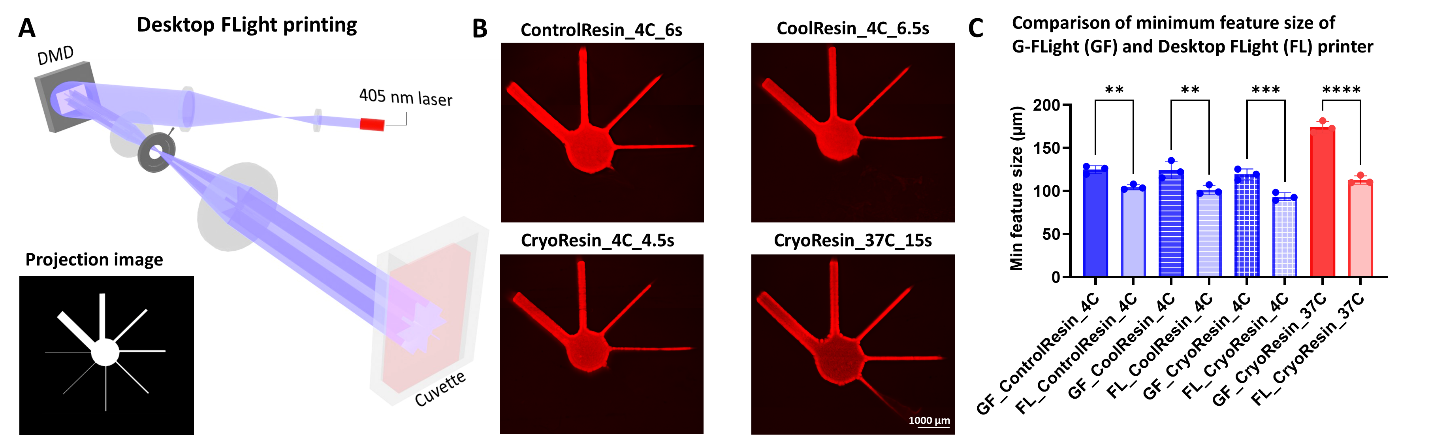


**Figure S8. A.** Illustration of the light path in the desktop FLight printer deploying a DMD for image control. **B.** Resolution images of the selected resin formulations printed using the FLight system. We used the same light dose as those for the G-FLight system. **C.** Comparison of the minimum feature sizes of the G-FLight (GF) and Desktop FLight (FL; DMD-based) system.


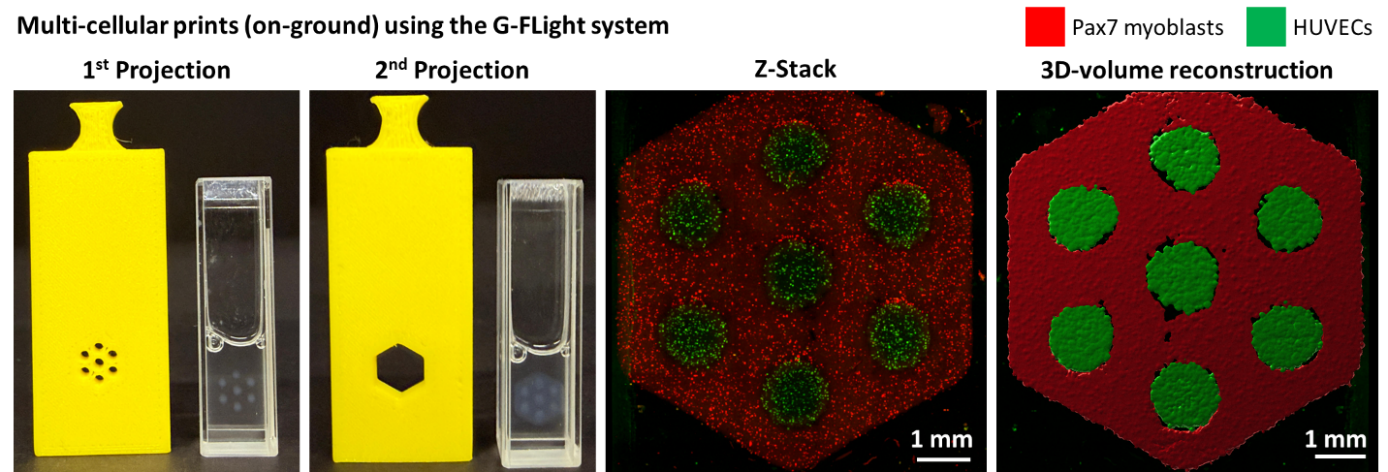


**Figure S9. Multicellular constructs printed on-ground (using CoolResin).** Briefly, glass cuvettes (4 mm path length) were filled with CoolResin formulations containing human umbilical vein endothelial cells (HUVECs; at 10^6^ cells mL^-1^) labelled with cell tracker green (Cytopainter, ab138891). Subsequently, an image pattern consisting of six circles was projected using a physical mask. Thereafter, the cuvette was gently washed using warm PBS to remove the uncrosslinked resin (the crosslinked constructs remained attached to the walls of the cuvettes). The cuvette was then refilled with fresh CoolResin containing Pax7 cells (at 10^6^ cells mL^-1^) labelled with cell tracker red (CMPTX Dye, C34552). A hexagonal image pattern was then projected using a different physical mask to crosslink the myoblast-laden resin around the preformed HUVEC-laden constructs, and the uncrosslinked resin washed away using warm PBS. The Z-stack image (obtained using confocal microscopy) and a 3D volume reconstruction of the crosslinked constructs is shown on the right.

**
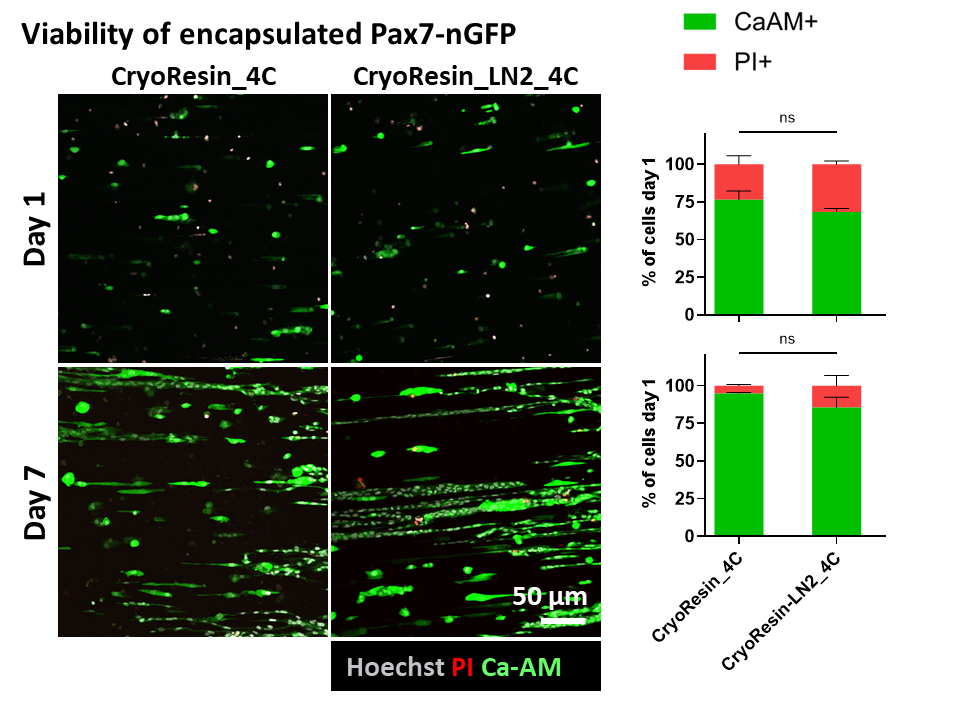
**

**Figure S10.** Viability of the Pax7-nGFP myoblasts in CryoResin formulations after storage in liquid N_2_ for over a week. There was no difference in the cell viability over a week between the CryoResin formulations stored at -80°C and those stored in liquid N_2_ (LN2 groups; -196°C), which is indicative of the potential of these resins to be used for long-term cryopresentation.


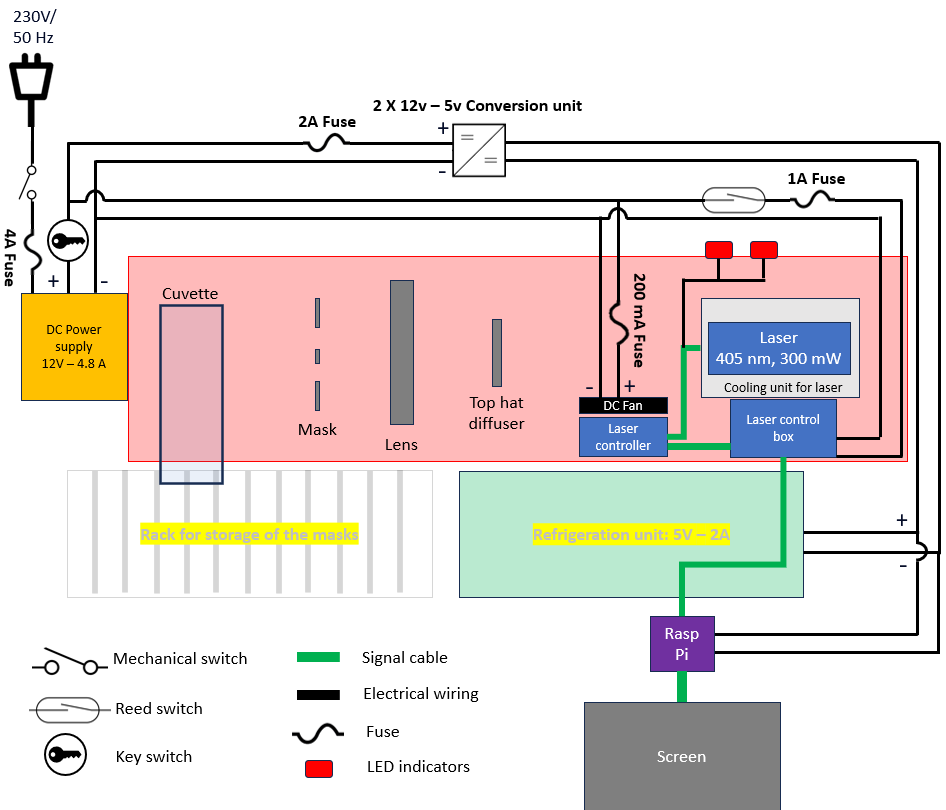


**Figure S11.** Component layout of the G-FLight system and corresponding electronic circuitry.


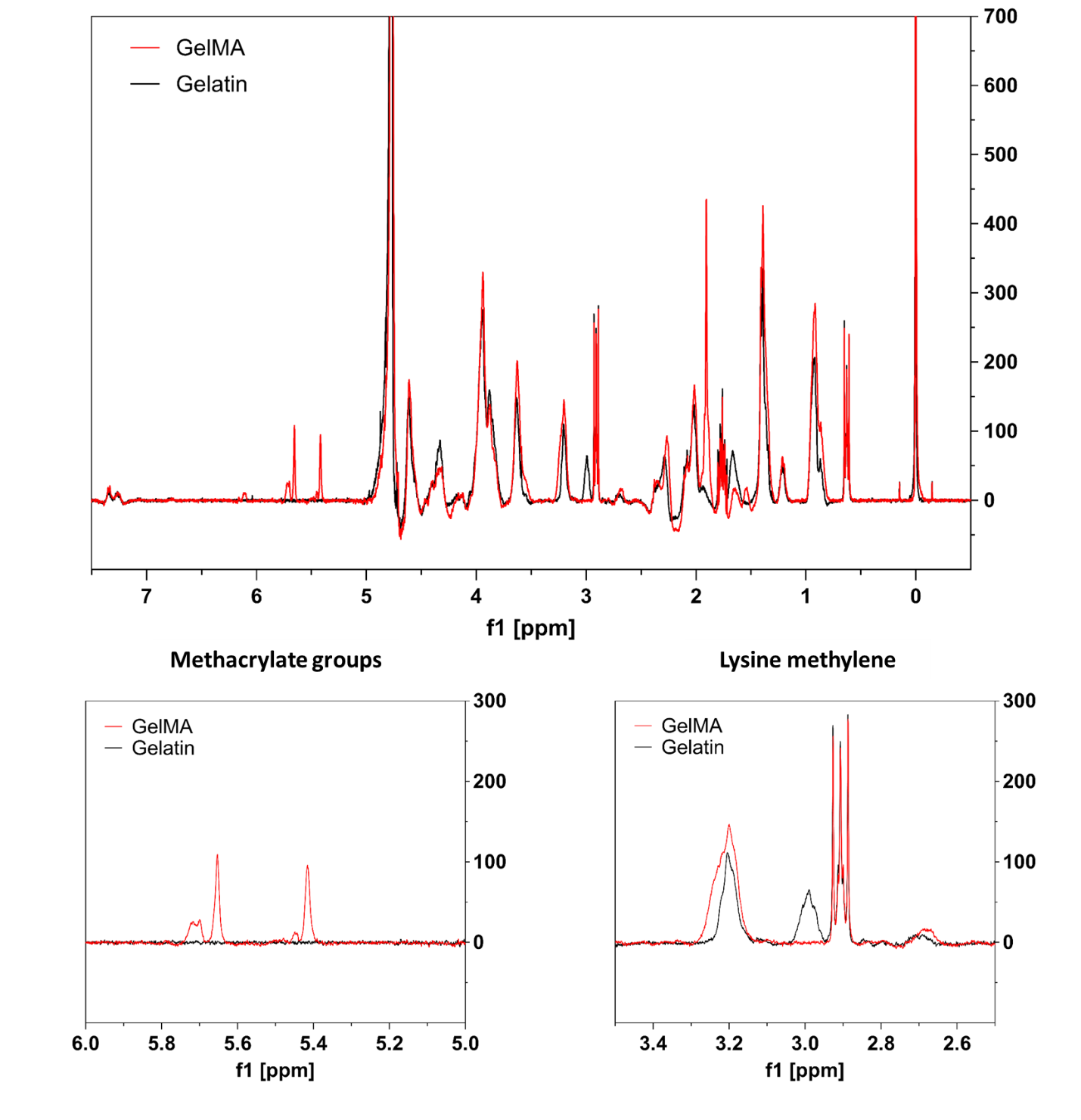


**Figure S12.** ^1^H NMR spectra of the GelMA formulation compared to pure gelatin.
